# A Polarized Discourse: Effects of Opinion Differentiation and Structural Differentiation on Communication

**DOI:** 10.1177/01461672211030816

**Published:** 2021-07-22

**Authors:** Namkje Koudenburg, Yoshihisa Kashima

**Affiliations:** 1 University of Groningen, The Netherlands; 2 University of Melbourne, Victoria, Australia

**Keywords:** polarization, communication, social structure, opinion-based groups, societal schism

## Abstract

In Western societies, many polarized debates extend beyond the area of opinions, having consequences for social structures within society. Such segmentation of society into opinion-based groups may hinder communication, making it difficult to reconcile viewpoints across group boundaries. In three representative samples from Australia and the Netherlands (*N* = 1,206), we examine whether perceived polarization predicts the quality (harmony, comfort, and experience of negative emotions) and quantity (avoidance of the issue) of communication with others in the community. We distinguish between perceived opinion differentiation (i.e., the extent to which opinions in society are divided) and perceived structural differentiation (i.e., the extent to which society fissions into subgroups). Results show that although opinion differentiation positively predicts the discussion of societal issues, the belief that these opinions reflect a deeper societal divide predicts negative communication expectations and intentions. We discuss how polarization perceptions may reinforce communicative behaviors that catalyze actual polarization processes.

In contemporary Western societies, many societal issues are subjected to an increasingly polarized discourse. At one level, the existence of different opinions is a healthy sign of democratic citizenry and a welcome expression of public engagement with a democratic polity (e.g., Cappella et al., 2002; Carpini et al., 2004; Gamson, 1992; McCoy et al., 2018; Mutz, 2006). Ideally in a democracy, ordinary citizens articulate their opinions and deliberate on opinion differences in their everyday conversation to address a societal issue of public interest (e.g., Jacobs et al., 2009). To this extent, opinion polarization should *encourage* people to talk about it. Yet, polarized debates may stifle public democratic engagement. This is because opinions may be so different that common ground may not be found (e.g., Kashima et al., 2007), making it difficult to communicate diverse viewpoints and inhibiting public deliberation (e.g., McCoy et al., 2018). Consequently, polarization may *discourage* a public expression of their opinions. Put crisply, when people believe that opinions are polarized on a societal issue, are they willing to bring it up in a conversation, or would they try to avoid it to reduce the risk of public confrontation? Past social psychological research suggests both are possible as our later review shows.

This article examines whether *perceived* polarization about a societal issue promotes or inhibits public deliberation. We define polarization as a state in which opinions in society are divided and partisan groups form around the divided opinions. In this sense, it differs from “group polarization” (Myers & Lamm, 1976). Critical in our definition is a distinction between perceived *opinion differentiation* (i.e., the extent to which opinions in society are perceived to be divided) and perceived *structural differentiation* (i.e., the extent to which society is seen to fission into subgroups rather than fusion into an integrated group), and we propose that although opinion differentiation may invite discussion of societal issues, the belief that these opinions are entrenched in subgroups in society and that there is a risk of societal fission may be detrimental for communication and deliberation. We develop this argument more fully below, generate specific hypotheses, and test them with three empirical studies conducted in the Netherlands and Australia.

## Opinion Differentiation and Communication

Abundant research suggests that most conversation is geared toward consensus. For instance, people prefer talking to others with similar opinions (e.g., Byrne, 1961), and in their conversations, people are more likely to discuss information that is shared among interaction partners than information that they uniquely hold (Clark & Kashima, 2007; Stasser & Titus, 1985). Such consensual communication serves both epistemic and relational human needs: It serves the belief that people have a shared, and therefore valid and reliable, understanding of the world around them while simultaneously increasing the experience of belonging to those who hold similar worldviews (Bar-Tal, 2000; Festinger, 1950; Hardin & Higgins, 1996). Because discussions tend to be geared toward reaching a common understanding of an issue, disagreements that threaten this understanding may lead to uncertainty (e.g., Moscovici & Personnaz, 1980). As a consequence, when opinion differences arise, this generally elicits some tension between members of a community (Schudson, 1997). Indeed, research shows that people are sensitive to subtle signals indicating that their opinions differ from those of others and experience them as threatening to both the consensus and the relationships with those others (Koudenburg et al., 2013a, 2017).

One strategy to reduce the tension due to opinion differences is by engaging in discussion. People direct 70% to 90% of their communication to group members with diverging opinions (Festinger & Thibaut, 1951; Schachter, 1951; see Wesselmann et al., 2014 for a recent replication), especially if they are expected to change their opinions (Festinger, 1950). In addition, there are informational benefits of discussing diverging opinions. In a healthy democracy, everyday political talk is a way for people to learn diverse political news and views, thereby increasing their understanding of others’ opinions and should therefore enhance tolerance of people with different political viewpoints (e.g., Cappella et al., 2002; Gamson, 1992; Mutz, 2006). Indeed, creating a shared reality through discussion may enhance a sense of understanding and control over one’s environment (Hardin & Higgins, 1996). People are especially motivated to develop a shared reality with others with whom they share a psychological group membership (S. A. Haslam et al., 1997; Kruglanski et al., 2006; Levine & Higgins, 2001).

## Social Structural Differentiation and Communication

A second strategy is to avoid the issue altogether (Noelle-Neumann, 1974; Wells et al., 2017). The foregoing provides a basis for considering under what circumstances deliberation about a societal issue may be avoided. It suggests that communication toward opinion deviants decreases when it becomes evident that they will not change their views (Schachter, 1951), or they are no longer seen to be members of the same psychological group (Festinger & Thibaut, 1951; Schachter, 1951). Put differently, when those with different opinions are seen to form different psychological groups, people may avoid talking about the topic that divides them. Such *opinion-based groups* (Bliuc et al., 2007; McGarty et al., 2009) develop when individuals who share similar opinions form a group although they might not have had any prior social relations and distinguish themselves from another group that is contrasted to their own group within the frame of reference defined by the superordinate society that includes both (Turner et al., 1987). Political parties are an obvious example, but the emergence of other opinion-based groups has been observed over the past decades (e.g., Alba & Foner, 2017; Dunlap et al., 2016).

Societal issues are at times discussed in terms of “us” (those who share our opinion) versus “them” (those with an opposing opinion; McCoy et al., 2018), thereby mutually rejecting each other’s viewpoints up to a point where a society seem to be fissioning into segregated opinion-based groups (Abramowitz & Saunders, 2008; McGarty et al., 2009; Shor & McCarty, 2011), prompting some to question the ability to talk to bridge political differences (Wells et al., 2017). The question is how opinion differentiation is transformed into opinion-based groups. Sani and Reicher’s (1998, 1999) work on schisms provides insights into this question. Their research on the split in the Church of England and in the Communist Party in Italy suggests that where opinion differences were seen to undermine the essence of the Church or the Party, they became non-negotiable—a source of division. Similarly, when people dynamically form contrasting opinion-based groups, people may come to believe that the shared opinion is the essence of each group, the groups have irreconcilably different essences, and there is a *structural differentiation* within society.

The belief about structural differentiation has significant consequences. First, essentialized opinion differences imply that those with different opinions cannot belong to the same group. Here, an opinion difference would signal a *relational threat*, which people would be motivated to avoid. Indeed, when engaging in communication with others in a community, it may be rewarding to maintain relationships superficially, without awareness of possible political differences (Macgregor, 2010). Second, when opinion differences are essentialized, opinions are likely believed to be hard to change. Cumulative research (Halperin et al., 2011; N. Haslam et al., 2006; Levy et al., 1998) has shown that people are more likely to stereotype essentialized social groups (Levy et al., 1998) and less willing to compromise in intergroup conflict (Halperin et al., 2011). By extrapolation, we expect that when people see structural differentiation, they would regard opinions to be unlikely to change and be less motivated to overcome differences through communication.

## Present Research: Perceived Polarization and Communication

The current research examines the psychological expectations associated with perceptions of polarization for everyday conversations within a community. We distinguished between perceptions of opinion differentiation and structural differentiation to test for their unique value in predicting expected quality (e.g., harmony and discomfort) and quantity (i.e., avoidance) of communication and experienced negative moral emotions. We hypothesized that both opinion and structural differentiations produce discomfort in communication, but that the strategies to alleviate this discomfort would be very different depending on the type of differentiation.

First, opinion differences may make a conversation less smooth and harmonious (*quality* of conversation; Koudenburg et al., 2017) because it threatens the assumption of consensus and shared reality and the conversants’ sense of belonging within the conversational group (Jans et al., 2019). The smooth flow of a conversation signals whether people are on the same wavelength: When conversations are smoothly flowing, they infer that they are probably in agreement on the issue at hand (Koudenburg et al., 2011, 2013a, 2017). Conversely, disrupted conversational flow is seen to signal a problem—either they lack consensus or their relationship is at risk. Hence, we expect that higher perceived opinion differentiation is likely associated with the expectation of lesser comfort and harmony in conversation (Hypothesis 1a). Moreover, expected discomfort is likely exacerbated when people perceive a deeper structural differentiation (Hypothesis 1b). Indeed, when disagreement threatens not only consensus but also social unity, a harmonious conversation is especially likely to be compromised.

Second, perceived opinion and structural differentiations on a societal issue are likely to have different implications for people’s willingness to discuss an issue or rather avoid it (*quantity* of conversation). On one hand, and in line with classic studies on social psychology (Festinger & Thibaut, 1951; Schachter, 1951) and political science (Gamson, 1992; Mutz, 2006), when people expect potential differences in opinion, they should be more willing to engage in discussion because they are motivated to reach a consensus on the issue or at least to increase understanding of the others’ viewpoints. Thus, perceptions of high opinion differentiation on a topic would increase people’s willingness to discuss it (Hypothesis 2a). Perceptions of structural differentiation, on the contrary, may result in an *avoidance* of the discussion about the issue. Avoidance is a common way to alleviate expected disagreement and discord on both the societal (Noelle-Neumann, 1974) and the interpersonal levels (MacKuen, 1990; Wells et al., 2017). With a greater risk of societal fission, we hypothesize that greater structural differentiation is associated with greater avoidance of the issue (Hypothesis 2b).

Third, opinion and structural differentiations on an issue may have different implications for *emotional experiences* if the topic is raised. We expect that mere opinion differences are unlikely to provoke negative moral emotions such as contempt, anger, and disgust. In fact, the awareness that multiple perspectives on the issue exist in society may relate to a less negative emotional response when encountering someone with a different perspective (Hypothesis 3a). However, when opinion differences are seen to structurally divide essentially different opinion-based groups, they are likely to predict negative moral emotions, signaling moral condemnation of those who hold “heretical” views (Hypothesis 3b; Haidt, 2003).

In three online studies using representative samples of the Australian (Study 1) and Dutch (Studies 2 and 3) population, we examined how discussion tendencies and expectancies were associated with their beliefs about opinion differentiation (Studies 1–3) and structural differentiation (Studies 2 and 3) in society. Study 3 additionally examined whether the processes of relational threat and incrementality beliefs could explain these relations. Data of all three studies is available at doi: 10.34894/XBV5VM.

## Study 1

Study 1 assessed whether perceived opinion polarization regarding several societal issues (e.g., refugees, gender roles, and carbon emissions) predicted the expected quality and quantity of the discussion and the expected negative moral emotions when these topics would come up (Hypotheses 1a, 2a, and 3a^1^). We examined perceived opinion polarization with a novel measure (Koudenburg et al., 2021; see also, Kusumi et al., 2017) but did not examine structural differentiation in this study.

### Method

#### Participants and design

Through *Qualtrics.Panels*, we employed a paid online sample, representative of the Australian population (*N* = 315, *M*_age_ = 45.3 years, *SD* = 16.7, range = 18–67, 49.5% female). Most participants had Australian nationality (71,1%), but the sample also included participants with a UK nationality (9.8%), New Zealand nationality (4.4%), or different nationalities (14.7%). Data of two participants who had no variance in their scores were removed before analysis. Power analyses were conducted to determine a sample size that would yield 80% power to detect a small effect. We used the effect size Cohen’s *W* (ω) for the change in χ^2^ when the Polarization Index would be added as a fixed factor to the model that already included all other variables. To correct for the interdependence of the data between repeated measures, we used the design effect (1 − ρ; Snijders & Bosker, 2011), estimating the correlation between measures (ρ) conservatively at .2 (higher correlations would result in more power). A ω of .1 is considered a small effect, .3 a medium effect, and .5 a large effect. With ω = .1, *df* = 1, α = .05, ρ = .2, we calculated that a sample size of 628 measurements (i.e., 314 participants) would be required for a test with a power of .80. This sample size would yield >99% power to detect a medium-sized effect.

The study had a repeated-measures design with two rounds. Although we were interested in the correlational results between polarization perceptions and communication tendencies, we introduced six different topics (two per participant) to (a) increase variation in polarization perceptions and (b) to ensure generalizability of the findings across different societal topics. Specifically, in Round 1, participants were randomly assigned to one of the three topics: refugees, gender roles, or acceptability of getting Botox (e.g., “Off-shore detention for refugees is unacceptable and must be stopped; it is inhumane to exclude people from society in this way”). In Round 2, they were randomly assigned to one of the three statements concerning the topic of carbon emission (e.g., “I would rather have nice and fancy appliances than energy-efficient ones”; see the wording of all statements in Supplemental Table A1). In each round, participants completed the same battery of measures.^2^

#### Polarization Index

To operationalize the perceived opinion polarization on the topic, participants were asked to distribute a random sample of 10 population representatives across five opinion categories (Koudenburg et al., 2021). Specifically, we asked them: *To statement X, how many out of 10 Australians do you think would strongly agree? How many out of 10 Australians do you think would agree?* And so on for *neither agree nor disagree/ disagree/ strongly disagree.* We constrained the responses so that the sum of these must be 10 (see Stimulus Materials).

We calculated a Polarization Index using a weighted sum of the level of disagreement between each pair of respondents from the distribution. Weights for the index were derived from the judgments of 60 polarization experts (see Koudenburg et al., 2021). The Polarization Index is calculated by *P* = 1.07 × % of score pairs (2,4) + 1.35 × % of score pairs (1,4)(2,5) + 1.98 × % of score pairs (1,5). Difference pairs (1,2)(2,3)(3,4)(4,5)(1,3)(3,5) receive no weight. The index is highly related to the standard deviation of the distribution (Study 1: *r* = .875, Study 2: *r* = .882, Study 3: *r* = .875) but only gives weight to pairs on different sides of the distribution and is therefore more sensitive to an exaggerated division between the two camps.^3^

#### Conversational harmony

Participants then indicated their conversation expectancies by imagining a conversation they would have at a neighborhood barbecue. We chose this context because (a) participants would not have strong a priori beliefs about their interaction partners’ opinions (at the neighborhood barbecue they could encounter any random person from the population although they likely have a similar sociodemographic profile) and (2) although relationships in this situation need not be very strong, participants likely expected to encounter their neighbors again at some point and therefore would be motivated to preserve the relationship.

In seven semantic differentials, participants indicated how they expected the conversation to be characterized when the topic would come up. Of these, three items measured conversational harmony: *conflicted* (1) *to consensual* (7), *uncomfortable* (1) *to comfortable* (7), *harmonious* (1) to *hostile* (7). The last item was reverse coded (Cronbach’s α = .779).

#### Avoidance

To assess avoidance behavior, we constructed a five-item scale (α = .830) consisting of one general avoidance item: “I would avoid this topic at the neighborhood barbecue”^4^ (1 = *not at all*, 7 = *very much*) and four items in which participants indicated whether two specific avoidant responses (avoiding/switching to a different topic and dropping a silence/looking away) to a target statement would be appropriate (1 = *very inappropriate*, 7 = *very appropriate*) and whether they would be likely to respond in this way (1 = *very unlikely*, 7 = *very likely*).

#### Personal attitude

To assess personal attitudes, participants indicated the extent to which they agreed with the target statement (1 = *not at all*, 7 = *very much*).

#### Negative emotions

Finally, participants indicated their levels of happiness, anger, comfort/at ease, surprise, indifference, disgust, and contempt after someone would express the target statement (1 = *not at all*, 7 = *very much*). A negative moral emotions scale was calculated from the items anger, disgust, and contempt (α = .843).

Table 1 provides descriptive statistics and correlations for all variables.

**Table 1.** Means, Standard Deviations, and Unilevel Correlations Between All Variables in Study 1.

	*M* (*SD*)	Pearson’s correlations			
Variable		2	3	4	5
1. Personal attitude	4.15 (2.05)	−.096*	.095*	−.058	−.242**
2. Polarization Index	0.27 (0.21)		−.124**	−.080*	−.089*
3. Conversational harmony	3.92 (1.35)			−.238**	−.186**
4. Avoidance	3.40 (1.48)				.270**
5. Negative emotions	2.78 (1.44)				

*Note.* Polarization Index scores range from 0 (*no polarization*) to 1 (*maximum polarization*). All other variables are measured on 7-point scales, with higher scores indicating higher agreement with the target statement (1), more anticipated conversational harmony (3), higher intentions to avoid (4), and more negative emotions in response to the target statement (5). *p < .05. **p < .01.

### Results

We conducted separate mixed-model regressions in SPSS to predict conversational harmony, avoidance, and negative emotions to a target statement (see Table 2). We included a random intercept and fixed effects for the predictor variables Polarization Index (unstandardized) and personal attitude (standardized with respect to grand mean and standard deviation of the full sample) and for the covariates topic, gender, and age. Because we had no a priori predictions about possible interaction effects of personal attitudes and the perceived polarization measures on the communication outcomes, we did not include these in any analyses. For the interested reader, we report the models including these interaction effects in the supplementary materials (Supplemental Appendix B). Crucially, in all three studies, the reported main effects of the perceived polarization measures remain statistically significant when including interaction terms in the model, pointing to the stability of the reported effects.^5^

**Table 2.** Parameter Estimates and 95% Confidence Intervals for each Dependent Variable in Study 1.

Predictor	Conversational harmony	Avoidance	Negative emotions
Intercept	4.50*** [3.08, 5.93]	3.38*** [1.63, 5,14]	3.18*** [1.59, 4.76]
[Topic=Botox]	−0.22 [−0.55, 0.10]	0.63*** [0.32, 0.96]	0.25 [−0.08, 0.57]
[Topic=Refugees]	−0.94*** [−1.27, −0.61]	0.82*** [0.50, 1.14]	0.45** [0.12, 0.78]
[Topic=Gender roles]	−0.73*** [−1.05, −0.40]	0.36* [0.04, 0.68]	0.41* [0.09, 0.74]
[Topic=Carbon 1]	−0.01 [−0.34, 0.34]	0.01 [−0.35, 0.33]	0.38* [0.03, 0.72]
[Topic=Carbon 2]	0.25 [−0.08, 0.59]	−0.22 [−0.56, 0.11]	−0.63*** [−0.97, −0.29]
[Topic=Carbon 3]			
[Gender=Male	−0.09 [−1.48, 1.30]	0.14 [−1.59, 1.86]	0.24 [−1.31, 1.79]
[Gender=Female]	−0.26 [−1.65, 1.13]	0.32 [−1.40, 2.05]	0.23 [−1.32, 1.78]
[Gender=Other]			
Age	−0.00 [−0.01, 0.01]	−0.02 [−0.01, 0.00]	−0.01** [−0.02, 0.00]
Z_Attitude	0.14** [0.04, 0.24]	−0.15** [−0.25, −0.05]	−0.36*** [−0.47, −0.26]
Polarization Index	−0.44† [−0.93, 0.05]	−0.74** [−1.27, −0.22]	−0.78** [−1.30, −0.27]

*Note.* Higher scores reflect more expected conversational harmony, higher intentions to avoid, and more expected negative emotions.

† *p* < .10. *p < .05. **p < .01. ***p < .001.

Personal attitude predicted all dependent variables, with higher agreement with the statement relating to increased expected conversational harmony, *t*(614) = 2.80, *p = .*005, reduced negative emotions in response to the target statement, *t*(614) = −6.95, *p* < .001, and reduced topic avoidance, *t*(614) = −2.91, *p* = .004.

The Polarization Index was related to the dependent variables in the hypothesized direction: Greater perceived opinion polarization predicted reduced conversational harmony, but this effect was only marginal: *t*(614) = −1.77, *p* = .078, ω = .07. However, it significantly predicted *reduced* negative emotions, *t*(614) = −3.00, *p = .*003, ω = .12 and *reduced* topic avoidance, *t*(614) = −2.78, *p* = .006, ω = .11.

### Discussion

A survey among an Australian community sample indicates that people’s expectancies and behavioral intentions regarding the discussion of societal issues depend not only on their personal attitude regarding that topic, but also on their perceptions of how polarized opinions on the issue are in society. First, and perhaps not surprisingly, the more people agree with a statement the more positive their expectancies regarding discussing this statement, and the less they intend to avoid the topic or experience negative emotions when it comes up.

Perceptions of opinion polarization also marginally significantly relate to more hostile, less harmonious conversation expectancies. Interestingly, however, the greater polarization people perceive in society, the less they intend to avoid the topic or anticipate negative emotions when it comes up. This suggests that when people perceive opinions as polarized, they may expect a certain level of tension when discussing the issue, but are also motivated to reach a common understanding. Moreover, because they are aware that opinion differences in society exist, they are not shocked to encounter someone with a different opinion.

## Study 2

Study 2 aimed to replicate Study 1 in a representative sample in the Netherlands. To increase understanding of how perceived polarization is conceived, we developed a novel measure to assess two aspects of polarization: *opinion differentiation* and *structural differentiation*. We expected the opinion differentiation subscale to be related to the numerical Polarization Index we employed in Study 1: It would assess to what extent opinions on the issue were divided within the population (and therefore served to test Hypotheses 1a, 2a, and 3a). The structural differentiation subscale aimed to measure the extent to which opinions on the issue were seen to be entrenched in different subgroups within society (to test Hypotheses 1b, 2b, and 3b).

In Study 2, we also addressed people’s emotional responses to statements that introduced the same issue but took an alternative position. It would be likely that one’s personal attitudes would elicit a more or less negative emotional response depending on the standpoint that was articulated. For opinion differentiation and structural differentiation, it might not matter whether the articulated view was pro or against a certain issue: The introduction of a controversial issue in itself would predict emotional responses, regardless of the position revealed by the statement.

### Method

#### Participants and design

We employed a paid online representative sample of the Dutch population recruited via *PanelInzicht.* Data of participants who had no variance in their scores (*n* = 5) or indicated not having the Dutch nationality (*n* = 5)^6^ were removed before analysis. The remaining sample included 460 participants^7^ (*M*_age_ = 49.7 years, *SD* = 17.2, range = 18–88, 50.7% female). Participants from all education levels were represented: 21% lower education, 40% middle education, 38% higher education. In all, 53% of participants was currently employed, 24% retired, 15% unemployed or volunteering, and 5% student.

The study had a repeated-measures design in which each participant was randomly offered two of four topics: refugees, income equality,^8^ Europe, or carbon emissions (see Supplemental Appendix Table A2 for the attitude and perceived polarization scores per statement). In each round, participants completed the same measures.

#### Measures

The survey included the same measures as Study 1 for personal attitude, the Polarization Index, avoidance (α = .72), and negative emotions to the target statement (α = .91). We added a fourth semantic differential to the conversational harmony scale: *smooth* (1) to *effortful* (7), reverse coded (α = .87). We also included two new measures: a polarization scale and a measure of emotional responses to a statement *opposing* the target statement.

##### Polarization scale

Participants indicated their agreement with eight statements on a 7-point scale. This Polarization Scale consisted of two subscales: *Opinion differentiation* and *Structural differentiation*, each consisting of the two items that reflected the core of these two constructs (see Table 3).^9^ Factor analysis with promax rotation was performed on each topic to not conflate within- and between-participant variance and to examine whether the same factor structure would apply to different topics. Both inspection of the scree plots and eigenvalue >1 criteria confirmed a two-factor structure for each of the topics, with opinion differentiation explaining 37.0% to 46.8% of the variance and structural differentiation explaining an additional 25.7% to 37.0% of the variance in the polarization scale. We tested for the similarity of factors across topics, and Tucker’s congruence coefficients ranged between .94 and .99, suggesting the factors were highly comparable across topics (Lorenzo-Seva & Ten Berge, 2006).

**Table 3.** Structure Matrix of Polarization Measure With Factor Loadings on Subscales Structural Differentiation and Opinion Differentiation.

Item	Opinion differentiation	Structural differentiation
*Opinion differentiation subscale*		
In the Netherlands, most people think the same about this issue.	[.86, .90]	[−.20, .02]
Although there may be some slight variations, most people share the same opinion on this issue.	[.85, .89]	[−.29, −.03]
*Structural differentiation subscale*		
Groups of people are in direct opposition of each other.	[−.37, .06]	[.78, .87]
There are subgroups forming in society that represent the different opinion camps.	[−.13, .07]	[.85, .87]

*Note.* Numbers in brackets indicate the range of factor loadings for the four different topics. Extraction method: principal component analysis. Rotation method: promax with Kaiser normalization.

##### Responses to the alternative statement

In addition to emotional reactions to the target statement, participants indicated their anticipated emotional reactions to an alternative statement on the same topic, which was formulated to voice the opinion opposite to the target statement. For instance, for a target statement “Asylum seekers should have access to the same facilities as other inhabitants of the Netherlands,” we phrased the alternative statement: “The Dutch should always be given priority at facilities in the Netherlands” (all statements are listed in Supplemental Appendix Table A2). This allowed us to test whether participants would respond emotionally to any statements regarding certain topics or only to those statements contrary to their views. The scores on anger, disgust, and contempt were combined into a scale (α = .91).

Table 4 provides descriptives and correlations for all variables.

**Table 4.** Means (Standard Deviations) of and Unilevel Correlations Between All Variables in Study 2.

	Pearson correlations							
Variable	*M* (*SD*)	2	3	4	5	6	7	8
1. Personal attitude	3.90 (1.77)	.065*	−.031	.067*	.115**	−.075*	−.309**	.300**
2. Polarization Index	0.32 (0.22)		.227**	.063	−.071*	−.090**	−.243**	−.104**
3. Opinion differentiation	4.32 (1.28)			.161**	−.249**	−.112**	−.196**	−.093**
4. Structural differentiation	4.55 (1.08)				−.293**	.172**	.116**	.133**
5. Conversational harmony	3.67 (1.17)					−.266**	−.129**	−.014
6. Avoidance	3.93 (1.10)						.295**	.119**
Negative emotions in response to:								
7. Target statement	2.86 (1.51)							.353**
8. Alternative statement	3.08 (1.56)							

*Note.* Polarization Index scores range from 0 (*no polarization*) to 1 (*maximum polarization*). All other variables are measured on 7-point scales, with higher scores indicating higher agreement with the target statement (1), larger perceived opinion differences (3), higher perceived structural differentiation (4), higher anticipated conversational harmony (5), higher intentions to avoid (6), and more anticipated negative emotions in response to the target statement (7) or a statement in opposition of the target statement (8).

Asterisks indicate that unilevel correlations are significant at *p < .05. **p < .01.

### Results

#### Polarization Index

To predict each dependent variable (i.e., conversational harmony, avoidance, and negative emotions to a target /alternative statement), we conducted four separate mixed-models regressions, including a random intercept, and fixed effects for the predictor variables personal attitude (standardized) and Polarization Index (unstandardized) and for the covariates topic, round (1 vs. 2), gender, age, living area (large city vs. small city vs countryside), and education (high vs. middle vs. low). Because living area worsened model fit in each model while explaining no significant variance in the outcome variables, we report the results of the mixed models excluding this covariate.

Table 5 displays the parameter estimates and 95% confidence intervals (CI) for the fixed effects. As expected, higher agreement with the statement (i.e., personal attitude) relates to greater expected conversational harmony, *t* = 4.41, *p* < .001, reduced topic avoidance, *t* = −2.48, *p* = .013, reduced negative emotions to the target statement, *t* = −11.36, *p* < .001, but increased negative emotions to the alternative statement, *t* = 10.69, *p* < .001.

**Table 5.** Parameter Estimates and 95% Confidence Intervals for the Polarization Index on Each Dependent Variable in Study 2.

Predictor	Conversational harmony	Avoidance	Negative emotions target statement	Negative emotions alternative statement
Intercept	3.94*** [3.63, 4.24]	3.90*** [3.58, 4.22]	4.00*** [3.60, 4.42]	3.57*** [3.14, 3.99]
[Topic=Refugees]	−0.33* [−0.59, −0.08]	−0.16 [−0.38, 0.05]	−0.17 [−0.48, 0.13]	0.28† [−0.04, 0.59]
[Topic=Carbon]	0.25* [0.01, 0.48]	−0.39*** [−0.58, −0.19]	−0.52*** [−0.80, −0.25]	−0.25† [−0.54, −0.04]
[Topic=Europe]	0.27* [0.01, 0.53]	−0.24* [−0.46, −0.03]	−0.57*** [−0.87, −0.27]	0.59*** [0.27, 0.91]
[Topic=Income]				
[Round=1]	−0.09 [−0.24, 0.06]	0.21*** [0.10, 0.32]	−0.02 [−0.18, 0.14]	−0.07 [−0.24, 0.09]
[Round=2]				
[Gender=male]	0.29*** [0.13, 0.44]	−0.01 [−0.18, 0.19]	0.44*** [0.22, 0.66]	0.29* [0.06, 0.52]
[Gender=female]				
[Education=low]	−0.01 [−0.23, 0.21]	0.08 [−0.17, 0.33]	0.27† [−0.04, 0.58]	0.10 [−0.22 0.42]
[Education=middle]	0.01 [−0.16, 0.19]	0.13 [−0.07, 0.34]	0.13 [−0.12, 0.38]	0.06 [−0.20 0.32]
[Education=high]				
Age	−0.01* [−0.01, −0.00]	0.00 [0.00, 0.01]	−0.01*** [−0.02, −0.01]	−0.01*** [−0.02, −0.01]
Z_Attitude	0.16*** [0.09, 0.23]	−0.08* [−0.14, −0.02]	−0.48*** [−0.57, −0.40]	0.48*** [0.39, 0.57]
Polarization Index	−0.43** [−0.76, −0.11]	−0.17 [−0.47, 0.14]	−1.16*** [−1.57, −0.75]	−0.61** [−1.04, −0.18]

† *p* < .10. *p < .05. **p < .01. ***p < .001.

The Polarization Index predicts three of the four dependent variables: In line with Hypotheses 1a and 3a, higher perceived polarization predicted reduced expected conversational harmony, *t* = −2.61, *p* = .009, ω = .09, and reduced negative emotional responses to both the target statement, *t* = −5.59, *p* < .001, ω = .18, and the alternative statement, *t* = −2.78, *p* = .006, ω = .09. We found no support for Hypothesis 2a; there was no significant relation with intentions to avoid the topic, *t* = −1.08, *p* = .278, ω = .04.

### Opinion Differentiation and Structural Differentiation

#### Relations between different Polarization Indices

We conducted two mixed-model analyses in which the Polarization Index (as a fixed factor) predicted the opinion differentiation and structural differentiation subscale. Both models included the covariates (topic, round, gender, education, and age) and personal attitude as fixed factors. The Polarization Index was related to the opinion differentiation subscale (*b* = 1.01, 95% CI = [0.72, 1.30], *t* = 6.79, *p* < .001) but not significantly related to the structural differentiation subscale (*b* = 0.23, 95% CI = [−0.06, 0.52], *t* = 1.58, *p* = .111).

#### Hypothesis testing

With another series of mixed-model regressions, we modeled the relations of the two polarization subscales (opinion differentiation and structural differentiation) and personal attitudes on the dependent variables. As in the previous analyses, we included the random intercept and the fixed effects of the predictors and of the covariates topic, round, gender, age, and education (see Table 6).

**Table 6.** Parameter Estimates and 95% Confidence Intervals for Perceived Opinion Differentiation and Structural Differentiation on Each Dependent Variable in Study 2.

Predictor	Conversational harmony	Avoidance	Negative emotions target statement	Negative emotions alternative statement
Intercept	3.80*** [3.52, 4.08]	3.86*** [3.55, 4.17]	3.71*** [3.32, 4.09]	3.42*** [3.01, 3.82]
[Topic=Refugees]	−0.18 [−0.43, 0.07]	−0.20† [−0.42, 0.02]	−0.14 [−0.45, 0.16]	0.27 [−0.05, 0.59]
[Topic=Carbon]	0.24* [−0.01, 0.47]	−0.36*** [−0.56, −0.17]	−0.44** [−0.72, −0.17]	−0.20 [−0.50, 0.09]
[Topic=Europe]	0.28* [0.04, 0.53]	−0.23* [−0.45, −0.02]	−0.53*** [−0.84, −0.23]	0.61*** [0.29, 0.92]
[Topic=Income]				
[Round=1]	−0.08 [−0.22, 0.06]	0.19*** [0.08, 0.0]	−0.06 [−0.22, −0.10]	−0.10 [−0.27, 0.07]
[Round=2]				
[Gender=male]	0.22** [0.07, 0.37]	0.00 [−0.18, 0.19]	0.37*** [0.15, 0.58]	.26* [0.03, 0.49]
[Gender=female]				
[Education=low]	−0.07 [−0.28, 0.13]	0.11 [−0.14, 0.36]	0.34* [0.04, 0.65]	0.15 [−0.17, 0.47]
[Education=middle]	−0.01 [−0.18, 0.15]	0.16 [−0.04, 0.36]	0.20 [−0.04, 0.44]	0.10 [−0.16, 0.36]
[Education=high]				
Age	−0.00* [−0.01, −0.00]	0.00 [−0.00, 0.01]	−0.02*** [−0.02, −0.01]	−0.01*** [−0.02, −0.01]
Z_Attitude	0.16*** [0.09, 0.23]	−0.09** [−0.14, −0.03]	−0.51*** [−0.60, −0.43]	0.46*** [0.37, 0.55]
Z_Opinion differentiation	−0.22*** [−0.29, −0.15]	−0.02 [−0.08, 0.05]	−0.24*** [−0.32, −0.15]	−0.11* [−0.20, −0.02]
Z_Structural differentiation	−0.26*** [−0.33, −0.19]	0.13*** [0.06, 0.20]	0.21*** [0.12, 0.30]	.17*** [0.07, 0.26]

† *p* < .10. *p < .05. **p < .01. ***p < .001.

As hypothesized, distinct patterns emerged for the polarization subscales. First, supporting Hypothesis 1, both opinion differentiation (Hypothesis 1a) and structural differentiation (Hypothesis 1b) were related to more hostile conversation expectancies (OD: *b* = −0.22, *t* = −6.41, ω = .21, *p* <. 001; *SD: b* = −0.26, *t* = −7.06, *p < .*001, ω = .23). No support was found for Hypothesis 2a: Perceived opinion differentiation did not predict avoidance behaviors, *b* = −0.02, *t* = −0.50, *p =* .621, ω = .02. Supporting Hypothesis 3a, opinion differentiation predicted reduced negative emotions when confronted with statements on either side of the opinion spectrum: target statement: *b* = −0.24, *t* = −5.40, *p < .*001, ω = .17; alternative statement: *b* = −0.11, *t* = −2.31, *p = .*021, ω = .08. However, perceptions of structural differentiation predicted *increased* avoidance of the topic (*b* = 0.13, *t* = 3.83, *p* < .001, ω = .12, supporting Hypothesis 2b) and more negative emotions when the topic was brought up, regardless of the position that was revealed (target statement: *b* = 0.21, *t* = 4.44, *p < .*001, ω = .14; alternative statement: *b* = 0.17, *t* = 3.35, *p < .*001, ω = .11, supporting Hypothesis 3b).

### Discussion

In Study 2, we replicated findings from Study 1 among a representative sample from the Dutch population: People’s expectancies and behavioral intentions when discussing societal issues are predicted by their personal attitude regarding that topic, but also on their perceptions of how polarized the issue is in society.

Interestingly, Study 2 demonstrated that personal attitudes only predicted increased negative moral emotions when people encountered an attitudinal statement diverging from one’s privately held attitude. In other words, personal attitudes had opposing consequences for the discussion of statements on different sides of the issue. However, perceptions of opinion differentiation and structural differentiation had similar consequences for discussion of statements on either side of the issue. That is, high perceived opinion differentiation predicted reduced negative emotions and reduced avoidance behaviors both when the statement articulated a view in line with or in contrast to the one held by the participant. This suggests that people are willing to discuss societal topics, likely to overcome potential differences. However, when differences are experienced as entrenched in societal subgroups, this willingness to discuss disappears: People intend to avoid the topic and feel disgusted, contemptuous, and angry when someone brings it up, regardless of the position this person takes on the issue.

## Study 3

**Figure 1.**
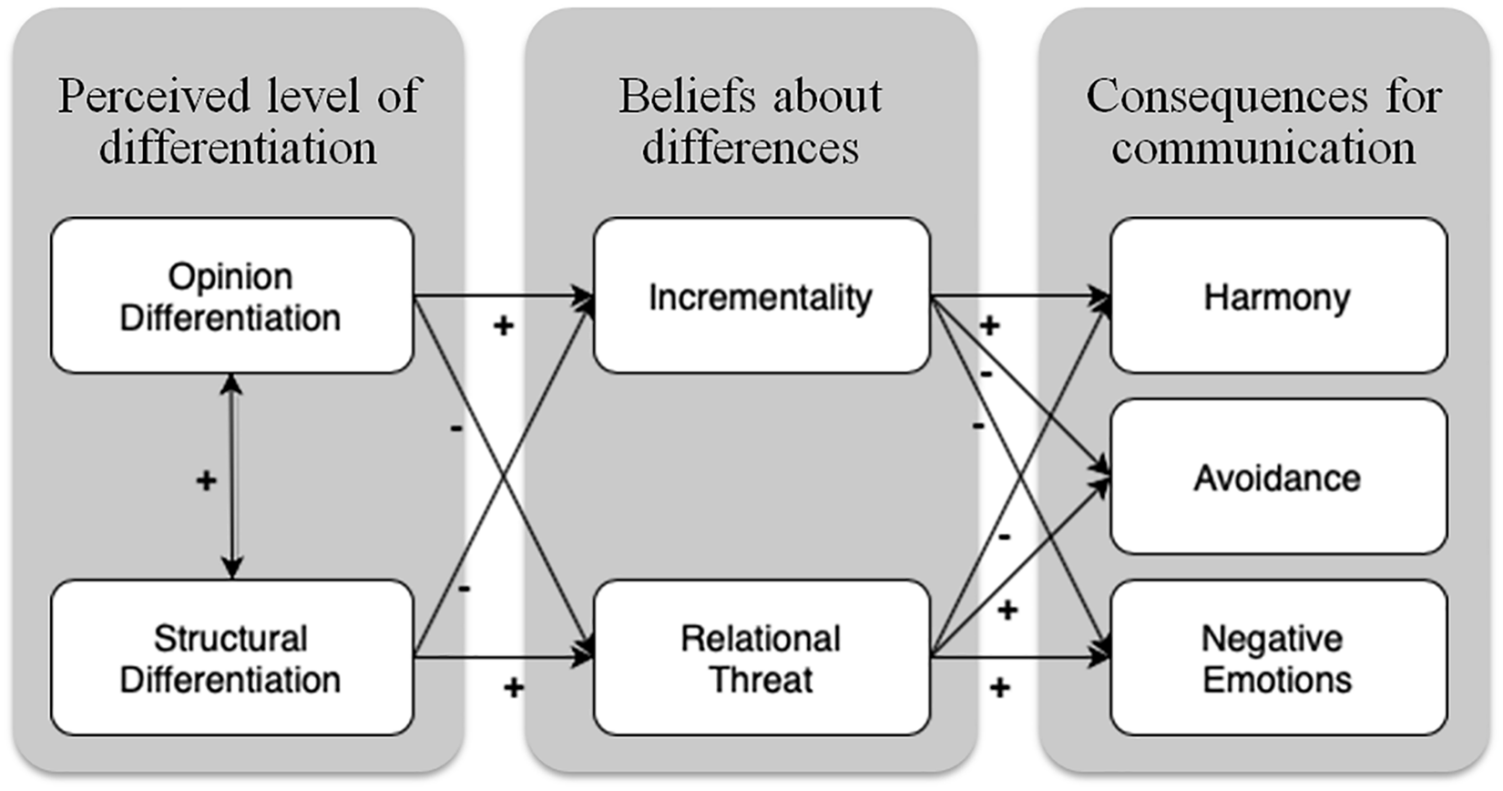
Hypothesized model.

Study 3 aimed to replicate the previous findings, with two additional aims. First, we aimed to test the causal relation between perceived opinion differentiation and conversational expectancies and behavioral intentions by manipulating perceptions of opinion differentiation. Second, we assessed two important process variables to explain why the levels of differentiation have different consequences for communication: relationship threat and incrementality of attitudes. We hypothesized that when people perceive differences to reflect deeper social divides (high perceived structural differentiation), they would be more likely to experience discussing these issues as threatening to the relationship (Hypothesis 4a) and unlikely to be effective in changing the other person’s attitudes (low incrementality, Hypothesis 4b), resulting in negative consequences for communication (Mediation Hypotheses 5a and 5b). In contrast, the awareness of opinion differences in society (high perceived opinion differentiation) may reduce the relational threat experienced when one encounters someone with a different opinion (Hypothesis 6a) and relate to beliefs that these are “just” opinions (Hypothesis 6b) that can be changed. Both these processes would result in positive consequences for communication (Mediation Hypotheses 7a and 7b). Please note here that although we predicted, in line with findings in Studies 1 and 2, a negative *direct* effect for opinion differentiation on conversational harmony, the *indirect* effect, through incrementality and relational threat, was hypothesized to be positive. For the full hypothesized model, see Figure 1.

### Method

#### Participants and design

A paid online sample representative of the Dutch population (*n* = 431, *M*_age_ = 48.91 *SD* = 17.95, range =18–95, 48% female, 51.5% male, 0.5% other)^10^ was recruited via *PanelInzicht*. All education levels were represented: 39% lower education, 32% middle education, 29% higher education. In all, 51% of participants was currently employed, 24% retired, 18% unemployed or volunteering, and 8% student.

The study had a repeated-measures design in which each participant was offered three opinion distribution conditions (polarized vs. dispersed vs. tight norm [either agreement or disagreement]) in random order.^11,12^ Each of the conditions informed participants about the opinion distribution on an opinion statement, which was randomly selected from a pool of 10 statements (see Stimulus Materials for the full list of statements) as recommended by Judd et al. (2012) to test generalizability across topics. In each round, participants completed the same measures.

#### Manipulation

In each condition, participants read a brief report about the outcomes of a study conducted at the University of Groningen. They were informed that 1,000 representatives of the Dutch population indicated the extent to which they agreed with a certain statement. The displayed results were different per condition. Participants in the high opinion differentiation condition read that people in the Netherlands were “very divided on the issue” or “had a diverse range of opinions on the issue” (both coded = 1), whereas people in the low opinion differentiation condition read that people in the Netherlands “mostly have similar opinions on the issue,” additionally stating “that people across the board disagreed with the statement” or “that people across the board, agreed with the statement” (both coded = 0).^13^

#### Measures

##### Dependent variables

The survey measured the dependent variables as in Study 1: topic avoidance (α = .69), conversational harmony (α = .83), and emotional responses to the target statement (α = .91).

##### Personal attitude

In addition to indicating their agreement with the target statement, we also exploratively included a measure of agreement with an alternative statement that was formulated in opposition of the target statement (see Methodology file for formulations of target and alternative statements).

##### Incrementality beliefs

Participants completed the three-item incrementality beliefs measure (Levy et al., 1998), adjusted to tap beliefs about opinion change on the specific issue, for example, “The opinions people have on this issue can’t be changed” (reverse coded, α = .85).

##### Relational threat

Participants indicated their anticipated relational threat in a three-item measure that we developed for the present study, for example, “*I* would feel distant if I find out the person I’m talking with has an opposing view on this issue” (α = .89).

#### Perceptions of polarization

Finally, participants completed the Polarization Index and both subscales of the Polarization Scale^14^: opinion differentiation (*r* = .50), structural differentiation (*r* = .39).

### Results

Table 7 provides descriptive statistics and correlations for all variables.

**Table 7.** Means (Standard Deviations) of and Unilevel Correlations Between All Variables in Study 3.

	*M* (*SD*)	Pearson’s correlations								
Variable		2	3	4	5	6	7	8	9	10
Personal attitude										
1. *Agreement target statement*	3.96 (2.04)	−.323**	−.019	−.142**	.084**	.027	.039	−.253**	.046	−.083**
2. *Agreement alternative statement*	3.77 (1.90)		−.054	−.149**	.100**	−.065*	.151**	.301**	.166**	−.133**
3. Polarization Index	0.34 (0.22)			.189**	.058*	−.002	−.092**	−.109**	−.053	.075**
4. Opinion differentiation	4.29 (1.33)				.097**	−.138**	−.166**	−.119**	−.213**	.228**
5. Structural differentiation	4.38 (1.24)					−.148**	.149**	.103**	.257**	−.243**
6. Conversational harmony	3.73 (1.27)						−.264**	−.140**	−.132**	.102**
7. Avoidance	3.65 (1.25)							.222**	.260**	−.287**
8. Negative emotions	3.01 (1.58)								.432**	−.149**
9. Relationship threat	2.93 (1.51)									−.266**
10. Incrementality	3.87 (1.29)									

*Note.* Polarization Index scores range from 0 (*no polarization*) to 1 (*maximum polarization*). All other variables are measured on 7-point scales, with higher scores indicating higher agreement with the target statement (1), higher agreement with the alternative statement (2), larger perceived opinion differences (4), higher perceived structural differentiation (5), higher anticipated conversational harmony (6), higher intentions to avoid (7), more anticipated negative emotions in response to the target statement (8), higher experienced relationship threat (9), and stronger beliefs that people can change their opinions about the issue (10).

Asterisks indicate that unilevel correlations are significant at *p < .05. **p < .01. ***p < .001.

#### Manipulation

The manipulation check revealed the intended effect on the opinion differentiation indicators, but the effects were rather small: Polarization Index: *b =* 0.03, *SE* = 0.01, *t* = 2.81, *p* = .005, 95% CI = [−0.05, −0.01], Cohen’s *d* = 0.12; opinion differentiation subscale, *b =* 0.25 *SE* = 0.06, *t* = 3.90, *p* < .001, 95% CI = [−0.37, −0.12], Cohen’s *d* = 0.19. Unintendedly, the manipulation also affected the structural differentiation subscale in the same direction: *b =* 0.19, *SE* = 0.06, *t* = 3.02, *p* = .003, 95% CI = [−0.31, −0.07], Cohen’s *d* = 0.15. Because the subscales should affect the outcome variables in opposite directions, it is possible that the effects canceled each other out, and therefore no effects on the other dependent variables appeared in this study (see Table 8).

**Table 8.** Means (Standard Deviations) Per Condition of Opinion Differentiation.

	Condition			
	Low opinion differentiation		High opinion differentiation	
Variable	Disagree (*n* = 210)	Agree (*n* = 209)	Dispersed (*n* = 418)	Polarized (*n* = 424)
Polarization Index	0.31 (0.23)	0.33 (0.21)	0.32 (0.21)	0.37 (0.23)
Opinion differentiation	4.16 (1.27)	4.07 (1.31)	4.36 (1.33)	4.38 (1.34)
Structural differentiation	4.29 (1.23)	4.22 (1.32)	4.43 (1.18)	4.43 (1.26)
Personal attitude				
Agreement target statement	3.80 (2.04)	4.25 (1.98)	3.84 (1.99)	4.01 (2.11)
Agreement alternative statement	3.84 (1.95)	3.62 (1.80)	3.81 (1.85)	3.76 (1.99)
Conversational harmony	3.79 (1.28)	3.82 (1.37)	3.72 (1.21)	3.71 (1.28)
Avoidance	3.75 (1.25)	3.60 (1.21)	3.65 (1.28)	3.60 (1.23)
Negative emotions	3.03 (1.63)	3.08 (1.59)	3.00 (1.54)	2.97 (1.56)
Incrementality	4.10 (1.24)	4.11 (1.27)	4.12 (1.25)	4.17 (1.35)
Relationship threat	2.90 (1.47)	3.06 (1.57)	2.93 (1.47)	2.89 (1.52)

#### Hierarchical linear modeling

Because the effect of the condition was small and did not affect the outcome variables, we conducted the remaining analyses similar to Study 1 and Study 2, by including the Polarization Index scores and the opinion differentiation and structural differentiation scales as continuous predictors in the analyses. We included condition as a categorical covariate in all analyses.

#### Relations between different polarization indices

Two mixed-models analyses were conducted as in Study 2. Both models included the covariates (round, gender, and age) and personal attitude as fixed factors, while topic was included as a random factor. The Polarization Index predicted scores on the opinion differentiation subscale (*b* = 0.75, 95% CI = [0.51, 1.00], *t* = 5.99, *p* < .001) and the structural differentiation subscale (*b* = 0.35, 95% CI = [0.10, 0.61], *t* = 2.78, *p* = .005).

#### Polarization index

We conducted mixed-model analyses for each of the five dependent variables (conversational harmony, avoidance, negative emotions, relationship threat, and incrementality; see Table 9). We included topic as a random factor (following guidelines by Judd et al., 2012) and other covariates as fixed factors in the model: condition (polarized vs. dispersed vs. agreement vs. disagreement), round (1 vs. 2 vs. 3), gender (male vs. female vs. other), and age.^15^ The personal attitudes (standardized) and the Polarization Index scores (unstandardized) were included as fixed effects in the model.

**Table 9.** Parameter Estimates and 95% Confidence Intervals for Polarization Index and the Personal Attitude on the DVs in Study 3.

Predictor	Conversational harmony	Avoidance	Negative emotions target statement	Relationship threat	Incrementality beliefs
Intercept	3.75*** [2.41, 5.08]	3.95*** [2.57, 5.34]	4.07*** [2.40, 5.73]	3.94*** [2.15, 5.75]	3.40*** [1.98, 4.82]
[R = 1]	0.14* [0.01, 0.28]	0.15* [0.02, 0.27]	−0.22** [−0.38, −0.07]	−0.08 [−0.21, 0.05]	0.23*** [0.11, 0.36]
[R = 2]	−0.07 [−0.21, 0.07]	0.04 [−0.08 0.16]	−0.07 [−0.23, 0.08]	−0.01 [−0.14, 0.13]	0.06 [−0.07, 0.19]
[R = 3]					
[C = polarized]	−0.11 [−0.28, 0.07]	0.04 [−0.12, 0.20]	−0.10 [−0.30, 0.10]	−0.11 [−0.28, 0.06]	−0.08 [−0.24, 0.09]
[C = dispersed]	−0.10 [−0.27, 0.08]	0.06 [−0.10, 0.22]	−0.11 [−0.31, 0.09]	−0.06 [−0.24, 0.11]	−0.02 [−0.19, 0.14]
[C = disagree]	−0.04 [−0.26, 0.17]	0.17† [−0.03, 0.37]	−0.10 [−0.35, 0.15]	−0.05 [−0.27, 0.17]	0.01 [−0.20, 0.22]
[C = agree]					
[G =male]	0.42 [−0.88, 1.73]	−0.75 [−2.11, 0.62]	−0.24 [−1.87, 1.39]	−0.53 [−2.30, 1.24]	0.56 [−0.84, 1.96]
[G =female]	0.26 [−1.05, 1.56]	−0.80 [−2.16, 0.57]	−0.39 [−2.01, 1.25]	−0.65 [−2.42, 1.11]	0.54 [−0.85, 1.94]
[G = other]					
Age	−0.01* [−0.01, −0.00]	0.01** [0.00, 0.01]	−0.01* [−0.01, −0.00]	−0.01* [−0.01, −0.00]	−0.01* [−0.01, 0.00]
Z_Attitude	−0.03 [−0.04, 0.09]	0.05 [−0.01, 0.11]	−0.44*** [−0.52, −0.36]	−0.02 [−0.09, 0.04]	−0.06† [−0.12, 0.01]
Polarization Index	−0.11 [−0.43, 0.20]	−0.34* [−0.64, −0.04]	−0.57** [−0.95, −0.21]	0.04 [−0.29, 0.39]	0.36* [0.02, 0.67]

† *p* < .10. *p < .05. **p < .01. ***p < .001.

Replicating Study 1, higher scores on the Polarization Index predicted a decreased intention to avoid the topic *b* = −0.34, *t* = −2.21, *p = .*027, ω = .06 (supporting Hypothesis 2a) and less anticipation of negative emotions when the topic would come up *b* = −0.58, *t* = −3.05, *p* = .002, ω = .08 (supporting Hypothesis 3a). In addition, the Polarization Index was related to higher incrementality beliefs, *b* = 0.36, *t* = 2.23, *p = .*026, ω = .06, suggesting that opinion differences go hand-in-hand with the expectation that people can change. We did not find evidence for a main effect for the Polarization Index on conversational harmony (Hypothesis 1a) or relationship threat, *t*s <.7, *p*s >.49.

#### Opinion differentiation and structural differentiation

We then created similar mixed models, including the same covariates, but this time including fixed effects for the standardized predictor variables personal attitude, perceived opinion differentiation, and perceived structural differentiation (see Table 10).^16^

**Table 10.** Parameter Estimates and 95% Confidence Intervals for the Subscales Opinion Differentiation and Structural Differentiation of the Polarization Questionnaire on Each Dependent Variable in Study 3.

Predictor	Conversational harmony	Avoidance	Negative emotions target statement	Relationship threat	Incrementality beliefs
Intercept	3.65*** [2.34, 4.95]	3.91*** [2.57, 5.25]	3.93*** [2.32, 5.55]	4.13*** [2.49, 5.77]	3.39*** [2.08, 4.68]
[R = 1]	0.19** [0.06, 0.33]	0.16** [0.04, 0.29]	−0.22** [−0.38, −0.07]	−0.06 [−0.20, 0.07]	0.20** [0.08, 0.33]
[R = 2]	−0.06 [−0.19, 0.08]	0.04 [−0.08 0.17]	−0.08 [−0.23, 0.08]	−0.00 [−0.13, 0.13]	0.05 [−0.08, 0.18]
[R = 3]					
[C = polarized]	−0.05 [−0.23, 0.12]	0.03 [−0.13, 0.19]	−0.13 [−0.33, 0.07]	−0.12 [−0.29, 0.06]	−0.07 [−0.24, 0.09]
[C = dispersed]	−0.04 [−0.21, 0.13]	0.06 [−0.10, 0.22]	−0.11 [−0.31, 0.09]	−0.08 [−0.25, 0.09]	−0.03 [−0.19, 0.14]
[C = disagree]	−0.02 [−0.24, 0.19]	0.17 [−0.03, 0.36]	−0.10 [−0.35, 0.14]	−0.07 [−0.29, 0.14]	0.02 [−0.19, 0.22]
[C = agree]					
[G =male]	0.41 [−0.88, 1.69]	−0.81 [−2.14, 0.51]	−0.28 [−1.87, 1.31]	−0.66 [−2.29, 0.97]	0.68 [−0.60, 1.97]
[G =female]	0.26 [−1.02, 1.54]	−0.82 [−2.14, 0.50]	−0.39 [−1.98, 1.20]	−0.73 [−2.36, 0.90]	0.61 [−0.68, 1.89]
[G = other]					
Age	−0.01* [−0.01, 0.00]	0.01*** [0.00, 0.01]	−0.01* [−0.01, 0.00]	−0.01** [−0.01, 0.00]	−0.00† [−0.01, 0.00]
Z_Attitude	0.02 [−0.05, 0.08]	0.02 [−0.04, 0.08]	−0.46*** [−0.53, −0.38]	−0.05 [−0.12, 0.01]	−0.02 [−0.08, 0.04]
Z_Opinion differentiation	−0.14*** [−0.21, −0.07]	−0.16*** [−0.23, −0.09]	−0.13** [−0.21, −0.05]	−0.23*** [−0.30, −0.16]	0.27*** [0.21, 0.34]
Z_Structural differentiation	−0.15*** [−0.22, −0.08]	0.15*** [0.08, 0.21]	0.17*** [0.09, 0.25]	0.31*** [0.23, 0.38]	−0.25*** [−0.31, −0.18]

*Note.* C = condition; R = round; G = gender.

† *p* < .10. *p < .05. **p < .01. ***p < .001.

#### Consequences for communication

Replicating the findings of Study 2, both opinion differentiation (*b* = −0.14, *t* = −3.98, *p* < .001, ω = .13) and structural differentiation (*b* = −0.15, *t* = −4.28, *p* < .001, ω = .14) predicted more hostile conversation expectancies (supporting Hypothesis 1a and 1b), but in line with Hypotheses 2a and 3a, only opinion differentiation predicted reduced avoidance of the topic (*b* = −0.15, *t* = −4.54, *p* <.001, ω = .15) and reduced negative emotions in response to the target statement (*b* = −0.13, *t* = −3.16, *p = .*002, ω = .10). Supporting Hypotheses 2b and 3b, structural differentiation perceptions predicted increased avoidance of the topic (*b* = 0.14, *t* = 4.25, *p* < .001, ω = .15) and increased negative emotions to the target statement (*b* = 0.17, *t* = 4.10, *p* < .001, ω = .13).

#### Processes

**Figure 2.**
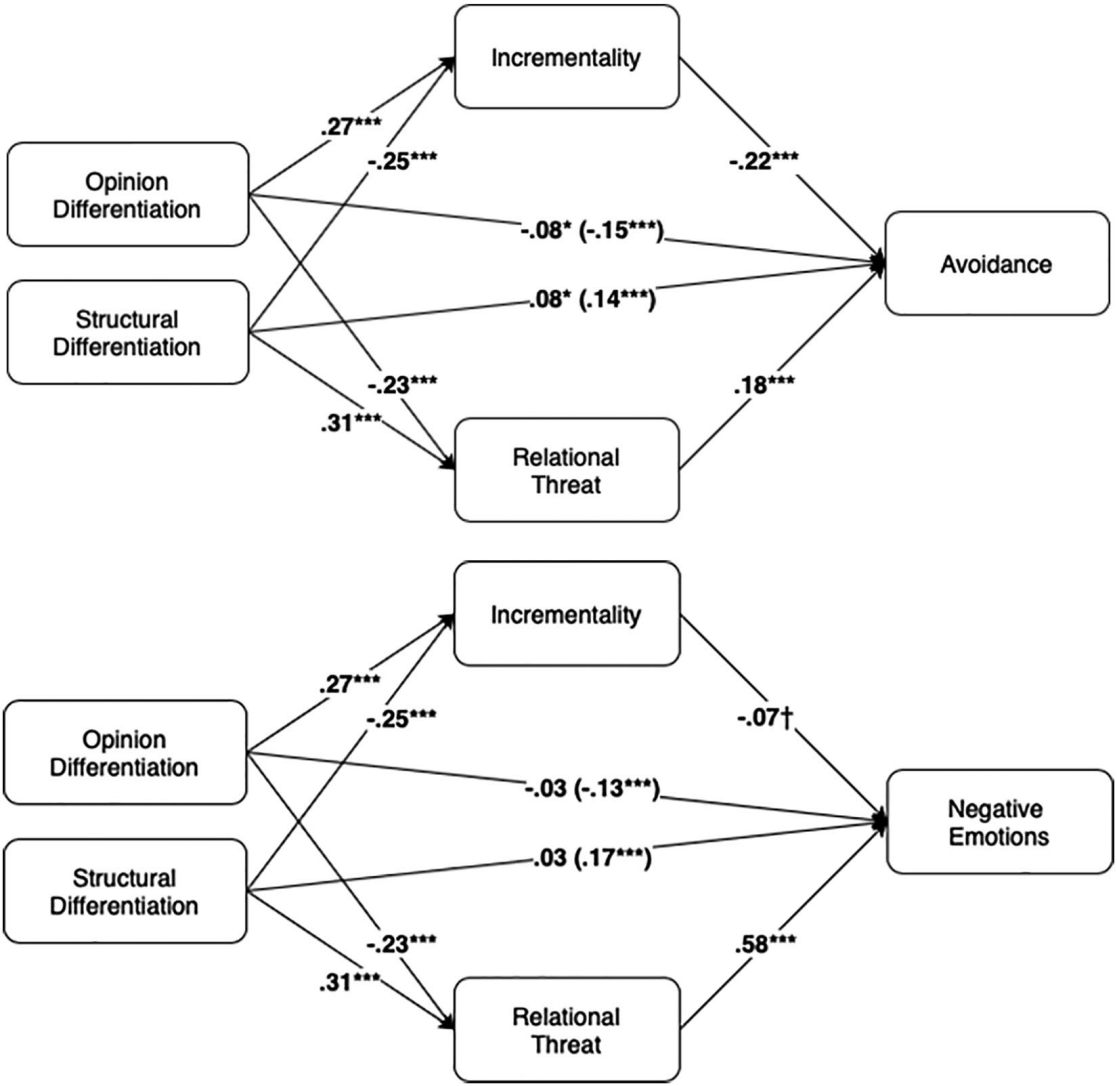
Mediation model of topic avoidance (Panel A) and negative emotions (Panel B).

To explain the distinct effects of the polarization subscales on communication tendencies, we examined whether relationship threat and incrementality mediated these effects. We used the guidelines by Krull and MacKinnon (2001) for multilevel mediation with all variables (X, M, and Y) measured at Level 1, nested within Level 2. We included random intercepts and fixed effects for the predictor variables. The a-paths were tested in two mixed-models analyses, in which opinion differentiation and structural differentiation were simultaneously included to predict incrementality/relationship threat. To assess the b-paths, three models specified the fixed effects of the standardized relationship threat and incrementality beliefs on each of the three communication tendencies, when opinion differentiation and structural differentiation were also in the models. Indirect effects were calculated by multiplying the multilevel beta coefficients for the a-paths (the effect of X on M) with the multilevel beta-coefficient for the b-paths (the effects of M on Y, when X was included in the model). The 95% CIs around these effects were calculated using the multilevel approximation for the standard error of the indirect effect (with the multivariate delta method/first-order Taylor series approximations, Sobel, 1986; recommended by Krull & MacKinnon, 2001). See Figure 2 for the models and multilevel regression coefficients.

The a-paths were as hypothesized: Structural differentiation predicted increased relationship threat (*b* = 0.30, *t* = 8.37, *p* < .001, ω = .27, Hypothesis 4a) and decreased incrementality beliefs (*b* = −0.25, *t* = −7.34, *p* < .001, ω = .23, Hypothesis 4b), while opinion differentiation predicted reduced relationship threat (*b* = −0.22, *t* = −6.12, *p* < .001, ω = .20, Hypothesis 6a), and increased incrementality beliefs (*b* = 0.27, *t* = 7.97, *p* < .001, ω = .26, Hypothesis 6b).

For the b-paths, relationship threat was significantly related to decreased conversational harmony, *b* = −0.16, *t* = −4.01, *p* < .001, 95% CI = [ 0.24, −0.08]; increased negative emotions, *b* = 0.58, *t* = 13.28, *p* < .001, 95% CI = [0.49, 0.66]; and increased avoidance, *b* = 0.18, *t* = 5.00, *p* < .001, 95% CI = [0.11, 0.26]. Similarly, incrementality beliefs were positively related to conversational harmony, *b* = 0.08, *t* = 2.15, *p* = .032, 95% CI = [0.01, 0.16], and decreased avoidance, *b* = −0.22, *t* = −6.36, *p* < .001, 95% CI = [−0.29, −0.15]. Incrementality beliefs were negatively related to negative emotions, but this effect was only marginal: *b* = −0.07, *t* = −1.74, *p =* .082, 95% CI = [−0.15, 0.01].

The CIs around the indirect effects show that, in line with Hypothesis 5a, relational threat mediated the effects of structural differentiation on negative emotions, *b* = 0.17, 95% CI = [0.12, 0.22], avoidance, *b* = 0.05, 95% CI = [0.03, 0.08], and conversational harmony, *b* = −0.05, 95% CI = [−0.07, −0.02]. Supporting Hypothesis 7a, relational threat significantly mediated the effects of opinion differentiation on negative emotions, *b* = −0.13, 95% CI = [−0.17, −0.08]; avoidance, *b* = −.04, 95% CI = [−0.06, −0.02]; and conversational harmony, *b* = −.03, 95% CI [0.01, 0.06]. Moreover, in partial support of Hypothesis 5b, incrementality beliefs significantly mediated the effects of structural differentiation on harmony, *b* = −0.02, 95% CI = [−0.04, −0.00], and avoidance, *b* = 0.06, 95% CI = [0.03, 0.08]. Similarly, supporting Hypothesis 7b, they mediate the effects of opinion differentiation on harmony, *b* = 0.02, 95% CI = [0.00, 0.04], and avoidance *b* = −0.06, 95% CI = [−0.08, −0.04]. We found no support for the Mediation Hypotheses on negative emotions (Hypotheses 5b and 7b). These indirect effects via incrementality beliefs were not significant (indirect effect of opinion differentiation: *b* = −0.02, 95% CI = [−0.04, 0.00] and of structural differentiation: *b* = −0.02, 95% CI = [−0.00, 0.04]).

Because of the correlational nature of the variables in the model, causality could be reversed. That is, highly emotional and avoidant communication may lead one to infer that opinion differences reflect structural divisions, whereas open and calm communication suggests mere opinion differentiation. Although this reasoning may be realistic (e.g., see our theorizing on the cyclical nature of the model in the discussion), the aim of the current research is to assess whether perceptions about differentiation can *influence* communication tendencies. Therefore, we also tested whether our manipulation would indirectly affect changes in the outcome variables by changing perceptions of opinion differentiation and structural differentiation.^17^ We conducted four unilevel serial indirect effects analyses to explore these additional paths. The full analyses and models are described in Supplemental Appendix C. We acknowledge that this mediation analysis does not take the multilevel data structure into full consideration and therefore may bias our parameter estimates to some extent. We interpret the results with caution.

Results show that our manipulation increased perceived opinion differentiation, which in turn decreased relational threat while increasing incrementality beliefs and, through that, increased anticipated harmony but decreased avoidance and negative emotions. Moreover, our manipulation increased structural differentiation, which in turn increased relational threat while decreasing incrementality beliefs and, through that, increased avoidance and negative emotions. The indirect paths of the manipulation via structural differentiation, through either incrementality or relational threat, on conversational harmony were not significant.

### Discussion

Study 3 replicates the findings of Study 2 by showing that both opinion differentiation and structural differentiation related to more hostile conversation expectancies (supporting Hypotheses 1a and 1b). Study 3 also provides additional support for Hypotheses 2a and 2b and Hypotheses 3a and 3b by showing that while opinion differentiation affects communication tendencies positively, the perception that such opinion differences reflect a deeper societal divide is detrimental for communication. Study 3 also offers an explanation for these findings by examining the processes of relationship threat and incrementality of beliefs. Specifically, results show that greater perceived opinion differentiation may strengthen beliefs that people may change their opinions and reduce relationship threat at the same time. Indirect effects analyses reveal that these processes may be able to explain the positive communication tendencies (higher expected harmony, reduced avoidance, and reduced negative emotions). By contrast, greater perceived structural differentiation may weaken beliefs that opinions can change and exacerbate relational threat, which could explain avoidance intentions and the anticipation of hostile conversation and negative emotions when the topic arises.

## General Discussion

Scientists and governments alike have been interested in discovering the reasons behind the increasing polarization in societies. In their explanations, opinion polarization has often been described interchangeably with the structural differentiation of society into (opinionated) subgroups (e.g., Hunter, 1991; Mouw & Sobel, 2001). However, the present article demonstrates that to understand processes of polarization it is necessary to distinguish these two levels of polarization because of their opposing implications for communication.

**Figure 3.**
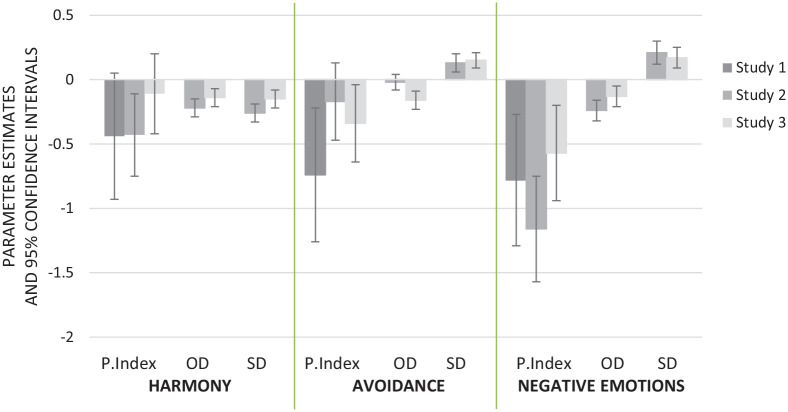
Summary of the results across studies. *Note.* Parameter estimates for Studies 1 to 3 of the effects of opinion differentiation, as indicated by the Polarization Index and opinion differentiation subscale, and of structural differentiation on anticipated conversational harmony and tendencies to avoid conversation and negative emotions when a topic were to come up. Error bars reflect 95% confidence intervals from the multilevel model. P.Index = Polarization Index; OD = Opinion differentiation subscale; SD = Structural differentiation subscale.

In three studies, we demonstrated that opinion differences elicit tension in everyday conversations with others in a community. In general, people expect conversations to be less harmonious and more uncomfortable when they perceive issues to be highly polarized. The present studies further demonstrated that there are different strategies to solve the discomfort that arises from opinion differences and that people are likely to base their strategy on the extent to which they expect differences to reflect a structural differentiation between groups in society. See Figure 3 for an overview of the findings across studies.

The first strategy is to engage in discussion. Converging evidence across three studies in Australia and the Netherlands shows that the greater opinion differences people perceive in the community, the *less* likely they are to avoid the topic when it is brought up. Moreover, they report anticipating *less* negative emotions (anger, disgust, and contempt) when controversial issues are brought up. These findings suggest that supporting the literature on common ground, and classic literature on social influence, people are willing to discuss different viewpoints to develop a shared reality (Hardin & Higgins, 1996), and such discussion is more required in situations in which viewpoints diverge (Festinger & Thibaut, 1951; Schachter, 1951).

Importantly, however, the current research identified a key boundary condition to this effect, explaining when people lose their motivation to discuss controversial issues but instead choose a strategy of avoiding the topic altogether. Specifically, Study 2 and 3 demonstrate that when people feel opinion differences on issues reflect a deeper structural divide between fundamentally different subgroups in society, they prefer to avoid the topic and experience negative moral emotions whenever it is brought up. In Study 3, we identified two key factors that help explain why people are motivated to engage in this strategy. When people perceive that opinion differences reveal a structural divide between groups, they are perceived as (a) entrenched and unlikely to be changed by discussion and (b) threatening a relationship, making people unlikely to be(come) friends. Therefore, to maintain the appearance of civility in the community, people may prefer not to delve deep into their opinion differences but rather refrain from discussing the issue (see also Macgregor, 2010).

Note that the effects of perceived polarization emerged above and beyond people’s personal attitudes. As many would predict (Byrne, 1961; Mutz, 2006), we found in three studies that people were more likely to engage with others that expressed an opinion similar to their own. Indeed, Study 2 demonstrated that personal attitudes had opposing consequences for the discussion of statements on different sides of the issue: People experienced more negative emotions when they encountered a statement opposing their attitude but experienced less negative emotions when encountering a statement that agreed with their own position. Importantly, this differs from the role of perceived opinion differentiation and structural differentiation: Here, we found *similar* consequences for discussion *regardless of which side people took*. That is, the greater is perceived opinion differentiation, the less is negative emotions and topic avoidance, regardless of whether the statement articulated a view in line with, or in contrast to, participants’ view. This suggests that people are willing to discuss societal topics and may overcome potential differences. However, when differences are seen to be entrenched in divided subgroups, the willingness to discuss disappears and a dread and avoidance sets in, feeling disgusted, contemptuous, and angry when someone brings it up, regardless of the position taken on the issue.

### Implications

The current research demonstrates that perceptions of polarization at different levels affect how people intend to behave in everyday conversations. This is important because social reality is shaped in these conversations such that people establish, maintain, and also change their reality by means of conversing with one another (Hardin & Higgins, 1996; Kashima et al., 2007). This implies that the perception of differences being reflections of a larger societal divide can produce conversational behaviors that could catalyze actual schisms in society. Specifically, we showed that perceptions of structural differentiation on specific issues lead people to expect the conversation to be uncomfortable and the opinion differences as hard to overcome and as a threat to the relationship. Consequently, they may feel disgusted and angry about the topic being brought up and likely try to avoid the issue by dropping a silence, looking away, or switching to a different topic. We know from previous research (Koudenburg et al., 2011, 2013b, 2017) that such behaviors may in fact communicate that relationships are in peril and as such threaten the social unity within the conversation group. In other words, perceptions that certain issues tear society apart may work as self-fulfilling prophecies by triggering communication behaviors that, in itself, disrupt social structures. As such, beliefs about polarization may catalyze social change.

That said, we note that perceiving opinion differences in itself does not appear to disrupt communication. In fact, much like how a democracy should function, opinion differences can invite healthy discussion. Such discussions could enhance understanding of different viewpoints, improve the coherence of opinion, and increase participation among citizens (e.g., Cappella et al., 2002; Habermas, 1985; Kim et al., 1999). This finding was obtained across two Western democracies: Australia and the Netherlands. Although these societies differ in some respects, they are both anchored in a strong democratic system (Norris, 2017), and democratic values are highly endorsed within the population as indicated by the World Value Survey (Inglehart et al., 2014; but see Norris, 2017). A different pattern might emerge in countries in which consensus is valued over open discussions, or decisions are not to be discussed because of high trust in authorities or, conversely, because deviant views are sanctioned. Future studies may examine the role of polarization perceptions across cultures.

Although part of our findings may be good news for democracy, they also suggest that the good news can turn sour when people believe that opinion differences reflect a deeper structural divide. But how do people come to believe a societal schism is emerging? Obviously, perceiving opinions to be divergent is not enough. It appears that the belief that opinions are entrenched in societal subgroups comes with an emotional component, a certain heatedness of discussion. We deem it therefore likely that the reverse may also occur: Perceiving heated discussions could be seen to imply a structural divide. With increasing hostility and emotiveness of political rhetoric in both traditional and social media, people are more likely to encounter emotional content (Geer, 2010; Iyengar et al., 2012), and such hostile and emotional content may further exacerbate polarization (Iyengar et al., 2012; Koudenburg et al., 2019). Indeed, even when actual attitudes remain constant, hostile discourse may promote categorization into groups with opposing views (Miller & Hoffmann, 1999). Current research suggests that through the belief that groups are fundamentally and “essentially” different, such hostile information streams can hamper everyday democratic discourse among citizens.

Opinion and structural differentiations are theoretically and empirically separable factors, which independently affect communication. Even when only a small minority of people disagrees with the majority on certain issues (i.e., low opinion differentiation), there could be variability in the extent to which this minority is seen as an opinion-based group that structurally diverges from the majority. Hence, although some level of opinion differentiation may be necessary for structural differentiation to be perceived, it need not be high, nor do we expect it to affect the effect of structural differentiation on communication. In line with this, the interaction effects between the two levels of polarization did not show a clear pattern across studies (see Supplementary Appendix B).

### Limitations

In the current research, we sought external validity by using multiple topics on which public opinions were divided and within two national contexts with samples from the Netherlands and Australia. However, we relied on self-reported behavioral intentions and correlational data. Future research should examine the generalizability of our findings to the actual conversation and the malleability of perceived opinion and structural differentiations.

## Conclusion

The present article demonstrates that to understand the consequences of perceptions of polarization for communication, one needs to distinguish between different levels of polarization. Specifically, when treating polarization as a difference of opinion, people do not show defensive reactions when encountering a different viewpoint. In fact, they experience less degrading moral emotions such as disgust and contempt but instead are motivated to overcome their differences. It is only when opinion differences are perceived as revealing deeper structural divisions that people come to believe that these opinions cannot be changed, and threaten their social relationships. As a result of these beliefs, people experience negative moral emotions when the topics come up and prefer to avoid discussion. As such, the perceptions that certain issues divide society into subgroups may work as a self-fulfilling prophecy by triggering communication behaviors that could catalyze actual societal polarization.

## Supplemental Material

sj-docx-1-psp-10.1177_01461672211030816 – Supplemental material for A Polarized Discourse: Effects of Opinion Differentiation and Structural Differentiation on Communication Click here for additional data file. Supplemental material, sj-docx-1-psp-10.1177_01461672211030816 for A Polarized Discourse: Effects of Opinion Differentiation and Structural Differentiation on Communication by Namkje Koudenburg and Yoshihisa Kashima in Personality and Social Psychology Bulletin

sj-docx-2-psp-10.1177_01461672211030816 – Supplemental material for A Polarized Discourse: Effects of Opinion Differentiation and Structural Differentiation on Communication Click here for additional data file. Supplemental material, sj-docx-2-psp-10.1177_01461672211030816 for A Polarized Discourse: Effects of Opinion Differentiation and Structural Differentiation on Communication by Namkje Koudenburg and Yoshihisa Kashima in Personality and Social Psychology Bulletin

## Notes

1 Prior to conducting Study 1, we hypothesized that opinion polarization would have negative consequences for discussion (i.e., elicit more negative emotions and lead to discussion avoidance). When effects appeared to be reversed, we developed the current model that differentiates between opinion differentiation and structural differentiation and tested this model in Study 2 and Study 3. To increase comprehension, we present only this final model in the case.

2 The studies were approved by the Ethical Committee Psychology of the University of Groningen with reference numbers 16037-O and 18076-0.

3 In addition to this measure, participants also selected one of the four graphical representations of opinion distributions and completed the same measures for the expected opinion distribution 10 years from now. Both measures were not analyzed for this article.

4 A second general item “I would like to discuss this topic at a neighborhood barbecue” reduced reliability of the scale and was therefore removed before further analyses.

5 An exception is the main effect of the Polarization Index on avoidance in Study 2, which is no longer significant when including the Polarization Index by attitude interaction in the model.

6 We included data of two participants who did not indicate their nationality.

7 With a correlation between repeated measures of *r* = .20, this sample would provide 92% power to detect a small effect of ω = .1.

8 Due to a randomization error, the topic income equality was never administered in the first round.

9 A factor analysis including all eight items produced the same two factors. We reduced the number of items by removing (a) the item: “People in the Netherlands are divided on the issue” which loaded on equally strong on both factors and (b) several items that were confounded with the process or outcome variables (e.g., they referred to opinions being entrenched or to communication tendencies). For a clearer interpretation of how structural differentiation predicts communication tendencies and beliefs about incrementality, we only included two core items in the analysis that concerned the grouping of people around certain opinions.

10 Data of eight non-Dutch participants was removed before analysis.

11 With a correlation between repeated measures of *r* = .20, this sample provided 98% power to detect a small effect of ω = .1.

12 Ten participants completed only two of the three conditions.

13 Table 8 displays the means of the four different phrasings. Because the core interest of this research is opinion differentiation and because the different phrasings within each condition did not cause differences in perceived opinion differentiation (or structural differentiation), we decided to not further differentiate those phrasings in the analyses.

14 Like in Study 2, we included the eight-item scale, but only focused on the 2 core items per subscale.

15 For none of the models, the fit worsened (compared to the empty model) by including these covariates, while for two of the five models fit improved significantly. For the sake of consistency, we included all covariates in all final models.

16 Including fixed effects for the predictor variables improved model fit (compared to the baseline model with only covariates) for all five modeled dependent variables.

17 We thank the editor for this suggestion.
