# Supplementary material for: A Polarized Discourse: Effects of Opinion Differentiation and Structural Differentiation on Communication
Source: Pers Soc Psychol Bull. 2021 Jul 22;48(7):1068–86. doi: 10.1177/01461672211030816 (PMC9178781; doi:10.1177/01461672211030816)
Supplement: sj-docx-1-psp-10.1177_01461672211030816 – Supplemental material for A Polarized Discourse: Effects of Opinion Differentiation and Structural Differentiation on Communication [file sj-docx-1-psp-10.1177_01461672211030816.docx]

**STUDY 1**

**Statements Round 1:**

**Refugees**

*“Off-shore detention for refugees is unacceptable and must be stopped; it is inhumane to exclude people from society in this way.”*

**Botox**

*“There is no reason to get all old and wrinkly, when we have Botox available to look as young as we want.”*

**Gender roles**

*"Women play a significantly greater part of fulfilling the caring role in our communities, which inevitably places some limits on their capacity in the workplace"*

**Statements Round 2:**

**Carbon emission**

*“I would rather have nice and fancy appliances than energy-efficient ones.”*

*“Everybody should be buying energy-efficient appliances to reduce their carbon footprint.”*

*“We need to have a society that doesn’t run on carbon emissions right now, otherwise we are doomed.”*

[Introduction Study]

**Polarization Index**

*Suppose that 10 people from all walks of life in Australia are asked to respond to an opinion survey. They are asked to say whether they would agree or disagree with a variety of opinions using a scale like below.*

*1 2 3 4 5*

*Strongly disagree disagree not agree/ or disagree agree strongly agree*

*To the statement: [XXX].*

*How many out of 10 do you think would strongly agree?*

*How many out of 10 do you think would agree?*

*How many out of 10 do you think would not agree or disagree?*

*How many out of 10 do you think would disagree?*

*How many out of 10 do you think would strongly disagree?*

*The total number of people must be 10.*

*[responses are constrained so that the sum of these must be 10]*

1. If you were to make a graph displaying the agreement and disagreement with this statement of people from Australia in 2016. What would this graph look like? Select 1 out of 5 graphs (see end of document).

Now imagine the same statement was made 10 years from now, in 2026. *Suppose that 10 different people from all walks of life in Australia are asked to say whether they would agree or disagree with this statement using the same scale. [present a scale]*

*To the statement: [XXX]*

*In 2026, how many out of 10 do you think would strongly agree?*

*In 2026, how many out of 10 do you think would agree?*

*In 2026, how many out of 10 do you think would not agree or disagree?*

*In 2026, how many out of 10 do you think would disagree?*

*In 2026, how many out of 10 do you think would strongly disagree?*

*The total number of people must be 10.*

1. If you were to make a graph displaying the agreement and disagreement with this statement of people from Australia in 2026? What would this graph look like? Select 1 out of 5 graphs (see end of document).

**Discussion Intentions [1]**

1. Imagine you are at a neighbourhood BBQ. After the first round of snacks and drinks, you engage in a conversation with some neighbours at your table. Suppose you would have to discuss two topics from the following list with the neighbours around your table. Which topic would you prefer to talk about?
   1. List: Off-shore detention of refugees, admission of refugees in Australia, gender roles in society, affirmative action policies, low-carbon emissions, global warming, acceptability of getting botox, healthcare allowance for plastic surgery.

About the target [not the chosen] topic:

1. I would like to discuss this topic at the neighbourhood BBQ (1 = not at all, 7 = very much).
2. I would avoid this topic at the neighbourhood BBQ (1 = not at all, 7 = very much).

**Conversational Expectations**

Imagine that the issue of [XXX] would come up at the neighbourhood BBQ, how do you expect that this conversation could be characterized?

1. Conflicted 1 2 3 4 5 6 7 Consensual
2. Calm 1 2 3 4 5 6 7 Aroused
3. Gentle 1 2 3 4 5 6 7 Coarse
4. Uncomfortable 1 2 3 4 5 6 7 Comfortable
5. Open 1 2 3 4 5 6 7 Closed / restricted
6. Harmonious 1 2 3 4 5 6 7 Hostile
7. Stilted 1 2 3 4 5 6 7 Flowing

**Discussion Intentions [2]**

Suppose you are at the neighbourhood BBQ, and someone expressed the statement: [XXX]

We would like you to think about the following responses to this statement and answer for each response: 1) how appropriate the response is, 2) how likely it is that you will respond in this way.

1. Explicitly expressing agreement with this statement
2. How appropriate do you think this response is?

(1 = very inappropriate, 7 = very appropriate)

1. How likely is it that you will act this way?

(1 = very unlikely, 7 = very likely)

1. Explicitly expressing disagreement with this statement
   1. How appropriate do you think this response is?
   2. How likely is it that you will act this way?
2. Avoid the topic or switch to a different topic.
   1. How appropriate do you think this response is?
   2. How likely is it that you will act this way?
3. Dropping a silence or looking away.
   1. How appropriate do you think this response is?
   2. How likely is it that you will act this way?

**Personal Attitude**

1. To what extent do you agree with the statement: XXX?

*Also add one or two distraction items. E.g., “If you read this question, fill out a 6.”

**Emotions**

Imagine the same conversation at the neighbourhood BBQ. To what extent would you experience the following emotions after someone would express the statement: [XXX].

Not at all very much

1. Happiness 1 2 3 4 5 6 7
2. Anger 1 2 3 4 5 6 7
3. Comfort/at ease 1 2 3 4 5 6 7
4. Surprise 1 2 3 4 5 6 7
5. Indifference 1 2 3 4 5 6 7
6. Disgust 1 2 3 4 5 6 7
7. Contempt 1 2 3 4 5 6 7

[Thank participant for participation]

**STUDY 2**

**Statements**

**Refugees**

*Target:* Asylum seekers should have access to the same facilities as other inhabitants of the Netherlands

*Alternative:* The Dutch should always be prioritized at facilities in the Netherlands.*.*

**Carbon Emissions**

*Target:* Everybody should eat less meat in order to save the environment

*Alternative:* Every human needs to eat at least one piece of meat a day.

**Europe**

*Target:* The Netherlands should leave the European Union.

*Alternative:* The EU should have more influence in the member states (including the Netherlands).

**Income**

*Target:* Rich people got where they are by working hard. They shouldn’t be punished for their wealth with higher tax rates.

*Alternative:* Many poor people ended up in poverty for reasons beyond their control. To help them, tax rates for the rich should be increased, and for the poor should be decreased.

**Demographics [1] and quota criteria**

Gender

Age

[Introduction Study]

**Polarization Index**

*Suppose that 10 people from all walks of life in Australia are asked to respond to an opinion survey. They are asked to say whether they would agree or disagree with a variety of opinions using a scale like below.*

*1 2 3 4 5*

*Strongly disagree disagree not agree/ or disagree agree strongly agree*

*To the statement: [XXX].*

*How many out of 10 do you think would strongly agree?*

*How many out of 10 do you think would agree?*

*How many out of 10 do you think would not agree or disagree?*

*How many out of 10 do you think would disagree?*

*How many out of 10 do you think would strongly disagree?*

*The total number of people must be 10.*

*[responses are constrained so that the sum of these must be 10]*

**Perceived Polarization Measure**

- In the Netherlands, most people think the same about this issue. (OD)
- People in the Netherlands are divided on the issue.
- There are subgroups forming in society that represent the different opinion camps. (SD)
- Although there may be some slight variations, most people share the same opinion on this issue. (OD)
- Groups of people are in direct opposition of each other. (SD)
- People’s opinions are not only divided, but also entrenched in the Netherlands.
- Exchanges between people with different opinions are getting more heated than before.
- People are getting fired up about their own views on this issue.

**Discussion Intentions [1]**

Imagine you are at a neighbourhood party. After the first round of snacks and drinks, you engage in a conversation with some neighbours.

Indicate to what extent you would like to discuss the topic [XXX] in such a conversation.

1. I would like to discuss this topic at the neighbourhood BBQ (1 = not at all, 7 = very much).
2. I would avoid this topic at the neighbourhood BBQ (1 = not at all, 7 = very much).

**Conversational Expectations**

Imagine that the issue of [XXX] would come up at the neighbourhood BBQ, how do you expect that this conversation could be characterized?

1. Conflicted 1 2 3 4 5 6 7 Consensual
2. Uncomfortable 1 2 3 4 5 6 7 Comfortable
3. Harmonious 1 2 3 4 5 6 7 Hostile
4. Smooth 1 2 3 4 5 6 7 Awkward

**Discussion Intentions [2]**

Suppose at the neighbourhood party someone expresses the statement: [XXX]

We would like you to think about the following responses to this statement and answer for each response: 1) how appropriate the response is, 2) how likely it is that you will respond in this way.

1. Explicitly expressing agreement with this statement
2. How appropriate do you think this response is?

(1 = very inappropriate, 7 = very appropriate)

1. How likely is it that you will act this way?

(1 = very unlikely, 7 = very likely)

1. Explicitly expressing disagreement with this statement
   1. How appropriate do you think this response is?
   2. How likely is it that you will act this way?
2. Avoid the topic or switch to a different topic.
   1. How appropriate do you think this response is?
   2. How likely is it that you will act this way?
3. Dropping a silence or looking away.
   1. How appropriate do you think this response is?
   2. How likely is it that you will act this way?

**Personal Attitude**

1. To what extent do you agree with the statement: [XXX]?

**Emotions**

Imagine the same conversation at the neighbourhood party. To what extent would you experience the following emotions after someone would express the statement: [XXX].

Not at all very much

1. Happiness 1 2 3 4 5 6 7
2. Anger 1 2 3 4 5 6 7
3. Comfort/at ease 1 2 3 4 5 6 7
4. Surprise 1 2 3 4 5 6 7
5. Indifference 1 2 3 4 5 6 7
6. Disgust 1 2 3 4 5 6 7
7. Contempt 1 2 3 4 5 6 7

To what extent would you experience the following emotions after someone would express the statement: [*Alternative statement XXX*].

Not at all very much

1. Happiness 1 2 3 4 5 6 7
2. Anger 1 2 3 4 5 6 7
3. Comfort/at ease 1 2 3 4 5 6 7
4. Surprise 1 2 3 4 5 6 7
5. Indifference 1 2 3 4 5 6 7
6. Disgust 1 2 3 4 5 6 7
7. Contempt 1 2 3 4 5 6 7

**Demographics [2]**

Nationality

Education

Employment status

Living in big city / small city / countryside

Province

[Thank participant for participation]

**STUDY 3**

|  | Target statement | Alternative statement |
| --- | --- | --- |
| **Refugees** | Asylum seekers should have access to the same facilities as other inhabitants of the Netherlands | The Dutch should always be prioritized at facilities in the Netherlands. |
| **Carbon Emission** | Everybody should eat less meat in order to save the environment | Every human needs to eat at least one piece of meat a day. |
| **Europe** | The Netherlands should leave the European Union. | The EU should have more influence in the member states (including the Netherlands). |
| **Income Inequality** | Rich people got where they are by working hard. They shouldn’t be punished for their wealth with higher tax rates. | Many poor people ended up in poverty for reasons beyond their control. To help them, tax rates for the rich should be increased, and for the poor should be decreased. |
| **#MeToo-discussion** | The #MeToo-discussion went way too far. Women are quick to speak of sexual intimidation. | The #MeToo-discussion is not sufficient. Sexual intimidation is a huge problem. |
| **Female leadership qualities** | Women have less good leadership qualities then men do. | Women have better leadership qualities than men do. |
| **Discouraging driving cars** | The Netherlands should increase taxes for driving cars, to discourage this. | There should be more roads in the Netherlands. |
| **Adoption for gays and singles** | Adoption should be available for everyone, including gay couples and singles. | Adoption should only be available for male-female couples. |
| **The importance of religion for society** | Religion does society more harm than good. | Religion is important for society. |
| **Costs of art** | Arts are an unnecessary cost for the government. | The government should invest more in arts. |

**Demographics [1] and quota criteria**

Gender

Age

[Informed Consent]

[Introduction Study]

**Manipulation Polarization**

In a recent study conducted at the University of XXX, a 1000 representatives of the Dutch population indicated the extent to which they agreed with a number of statements. In this study, we would like to share their responses with you. Afterwards, you will be asked a number of questions on each of these statements.

You will now see the results for the statement [XXX}

***High opinion differentiation conditions***

[1] The results of the study show that the Dutch are very divided on this issue. About half of the respondents disagreed or strongly disagreed with the statement, the other half agreed or strongly agreed. Only a few respondents appeared to be neutral on the subject. The graph below depicts the results of the study.

[2] The results of the study show that the Dutch had a diverse range of opinions on the issue. There are respondents who agree, some stronger than others. There are also respondents who disagree, some stronger than others, and there are respondents who take a neutral position on the issue. The graph below depicts the results of the study.

**Low opinion differentiation conditions**

[1] The results of the study show that most Dutch think in the same way about the issue. Most respondents agree with the statement. Although there are subtle differences, across the board, respondents share this opinion. The graph below depicts the results of the study.

[2] The results of the study show that most Dutch think in the same way about the issue. Most respondents disagree with the statement. Although there are subtle differences, across the board, respondents share this opinion. The graph below depicts the results of the study.

**Discussion Intentions [1]**

Imagine you are at a neighbourhood party. After the first round of snacks and drinks, you engage in a conversation with some neighbours.

Indicate to what extent you would like to discuss the topic [XXX] in such a conversation.

1. I would like to discuss this topic at the neighbourhood BBQ (1 = not at all, 7 = very much).
2. I would avoid this topic at the neighbourhood BBQ (1 = not at all, 7 = very much).

**Conversational Expectations**

Imagine that the issue of [XXX] would come up at the neighbourhood BBQ, how do you expect that this conversation could be characterized?

1. Conflicted 1 2 3 4 5 6 7 Consensual
2. Uncomfortable 1 2 3 4 5 6 7 Comfortable
3. Harmonious 1 2 3 4 5 6 7 Hostile
4. Smooth 1 2 3 4 5 6 7 Awkward

**Discussion Intentions [2]**

Suppose at the neighbourhood party someone expresses the statement: [XXX]

We would like you to think about the following responses to this statement and answer for each response: 1) how appropriate the response is, 2) how likely it is that you will respond in this way.

1. Explicitly expressing agreement with this statement
2. How appropriate do you think this response is?

(1 = very inappropriate, 7 = very appropriate)

1. How likely is it that you will act this way?

(1 = very unlikely, 7 = very likely)

1. Explicitly expressing disagreement with this statement
   1. How appropriate do you think this response is?
   2. How likely is it that you will act this way?
2. Avoid the topic or switch to a different topic.
   1. How appropriate do you think this response is?
   2. How likely is it that you will act this way?
3. Dropping a silence or looking away.
   1. How appropriate do you think this response is?
   2. How likely is it that you will act this way?

**Personal attitude + attitude alternative statement**

1. To what extent do you agree with the statement: [XXX]?
2. To what extent do you agree with the statement: [Alternative XXX]?

**Emotions**

Imagine the same conversation at the neighbourhood party. To what extent would you experience the following emotions after someone would express the statement: [XXX].

Not at all very much

1. Happiness 1 2 3 4 5 6 7
2. Anger 1 2 3 4 5 6 7
3. Comfort/at ease 1 2 3 4 5 6 7
4. Surprise 1 2 3 4 5 6 7
5. Indifference 1 2 3 4 5 6 7
6. Disgust 1 2 3 4 5 6 7
7. Contempt 1 2 3 4 5 6 7

**Incrementality beliefs**

To what extent do you agree with the following statements about topic [xxx]. (1 = not at all, 7 = very much).

1. The opinions people have on this issue can't be changed.
2. People can do or say things differently, but the important parts of what they believe about this issue can't really be changed.
3. Everyone thinks in a certain way about this issue, and there is not much that one can do about that.

**Relational threat**

1. It is difficult to make friends with someone who has a different view on this issue
2. If someone disagrees with me on this issue, this person can never be my friend
3. I would feel distant if I find out the person I’m talking with has an opposing view on this issue

**Polarization Index**

*Suppose that 10 people from all walks of life in Australia are asked to respond to an opinion survey. They are asked to say whether they would agree or disagree with a variety of opinions using a scale like below.*

*1 2 3 4 5*

*Strongly disagree disagree not agree/ or disagree agree strongly agree*

*To the statement: [XXX].*

*How many out of 10 do you think would strongly agree?*

*How many out of 10 do you think would agree?*

*How many out of 10 do you think would not agree or disagree?*

*How many out of 10 do you think would disagree?*

*How many out of 10 do you think would strongly disagree?*

*The total number of people must be 10.*

*[responses are constrained so that the sum of these must be 10]*

**Perceived Polarization Measure**

- In the Netherlands, most people think the same about this issue. (OD)
- People in the Netherlands are divided on the issue.
- There are subgroups forming in society that represent the different opinion camps. (SD)
- Although there may be some slight variations, most people share the same opinion on this issue. (OD)
- Groups of people are in direct opposition of each other. (SD)
- People’s opinions are not only divided, but also entrenched in the Netherlands.
- Exchanges between people with different opinions are getting more heated than before.
- People are getting fired up about their own views on this issue.

**Demographics [2]**

Nationality

Education

Employment status

Living in big city / small city / countryside

Province

[Thank participant for participation]
