# Supplementary material for: A Polarized Discourse: Effects of Opinion Differentiation and Structural Differentiation on Communication
Source: Pers Soc Psychol Bull. 2021 Jul 22;48(7):1068–86. doi: 10.1177/01461672211030816 (PMC9178781; doi:10.1177/01461672211030816)

**Appendix A. Means attitude ratings and polarization perceptions per topic in each Study.**

Table A1. Means and standard deviations of the predictor variables per topic in Study 1. Personal attitudes reflect the extent to which participants agreed with the target statement (1 = not at all, 7 = very much). Polarization index scores range from 0 (no polarization) to 1 (maximum polarization).

Table A2. Means (standard deviations) of the predictor variables per topic in Study 2. Personal attitudes reflect the extent to which participants agreed with the target statement (1 = not at all, 7 = very much). Polarization index scores range from 0 (no polarization) to 1 (maximum polarization). Opinion differentiation and structural differentiation scores range from 1 to 7, with higher scores indicating larger perceived differences.

**Appendix B. Analyses including interaction effects for all three studies.**

Table B1. Parameter estimates and 95% confidence intervals for the effects of the polarization index, personal attitude (Att) and their interaction on the DVs in Study 1 (Australian Sample).

|  | Conversational harmony | | | Avoidance | | | Negative emotions target statement | | |
| --- | --- | --- | --- | --- | --- | --- | --- | --- | --- |
| Intercept | 4.48*** | [3.07; | 5.90] | 3.38*** | [1.62; | 5.14] | 3.15*** | [1.57; | 4.75] |
| [Topic=Botox] | -0.20 | [-0.53; | 0.12] | 0.64*** | [0.33; | 0.97] | 0.26 | [-0.06; | 0.59] |
| [Topic=Refugees] | -0.91*** | [-1.23; | -0.58] | 0.84*** | [0.51; | 1.16] | 0.47** | [0.14; | 0.80] |
| [Topic=Gender roles] | -0.72*** | [-1.05; | -0.40] | 0.37* | [0.05; | 0.68] | 0.42* | [0.09; | 0.74] |
| [Topic=Carbon_1] | 0.03 | [-0.31; | 0.37] | 0.01 | [-0.33; | 0.35] | 0.40* | [0.06; | 0.74] |
| [Topic=Carbon_2] | 0.27 | [-0.06; | 0.61] | -0.21 | [-0.55; | 0.12] | -0.62*** | [-0.96; | -0.28] |
| [Topic=Carbon_3] |  |  |  |  |  |  |  |  |  |
| [Gender=Male | -0.08 | [-1.46; | 1.30] | 0.14 | [-1.58; | 1.87] | 0.25 | [-1.31; | 1.81] |
| [Gender=Female] | -0.24 | [-1.63; | 1.14] | 0.33 | [-1.40; | 2.05] | 0.25 | [-1.31; | 1.81] |
| [Gender=Other] |  |  |  |  |  |  |  |  |  |
| Age | 0.00 | [-0.01; | 0.01] | 0.00 | [-0.01; | 0.00] | -0.01** | [-0.02; | -0.00] |
| Polarization Index | -0.47† | [-0.96; | 0.01] | -0.76** | [-1.28; | -0.23] | -0.80** | [-1.31; | -0.28] |
| Z_Att | 0.28*** | [0.13; | 0.44] | -0.20 | [-0.26; | 0.06] | -0.26** | [-0.42; | -0.10] |
| Polarization Index * Z_Att | -0.51* | [-0.95; | -0.07] | -0.20 | [-0.65; | 0.24] | -0.36 | [-0.81; | 0.08] |

*** *p* < .001, ** *p* < .01, * *p* < .05, † *p* < .10

Table B.2.1 Parameter estimates and 95% confidence intervals for the effects of subscales Opinion differentiation (OD) and Structural differentiation (SD) of the polarization questionnaire, personal attitudes (Att), and their interactions on each DV in Study 2 (Dutch Sample).

|  | Conversational harmony | | | Avoidance | | | Negative emotions  target statement | | | Negative emotions alternative statement | | |
| --- | --- | --- | --- | --- | --- | --- | --- | --- | --- | --- | --- | --- |
| Intercept | 3.79*** | [3.51 | 4.06] | 3.84*** | [3.54 | 4.14] | 3.71*** | [3.33 | 4.08] | 3.40*** | [3.00 | 3.80] |
| [Topic=Refugees] | -0.18 | [-0.43 | 0.06] | -0.17 | [-0.38 | 0.04] | -0.10 | [-0.41 | 0.20] | 0.30† | [-0.02 | 0.62] |
| [Topic=Carbon] | 0.22† | [-0.01 | 0.44] | -0.35*** | [-0.54 | -0.16] | -0.46** | [-0.74 | -0.19] | -0.18 | [-0.48 | 0.11] |
| [Topic=Europe] | 0.25* | [0.00 | 0.49] | -0.25* | [-0.46 | -0.05] | -0.57*** | [-0.87 | -0.26] | 0.61*** | [0.29 | 0.93] |
| [Topic=Income] |  |  |  |  |  |  |  |  |  |  |  |  |
| [Round=1] | -0.06 | [-0.19; | 0.08] | 0.20*** | [0.10 | 0.31] | -0.05 | [-0.21 | -0.12] | -0.11 | [-0.28 | 0.06] |
| [Round=2] |  |  |  |  |  |  |  |  |  |  |  | . |
| [Gender=male] | 0.21** | [0.06; | 0.36] | -0.01 | [-0.18 | 0.17] | 0.35** | [0.14 | 0.56] | .25* | [0.03 | 0.48] |
| [Gender=female] |  |  |  |  |  |  |  |  |  |  |  |  |
| [Education=low] | -0.08 | [-0.29 | 0.13] | 0.08 | [-0.17 | 0.32] | 0.29* | [0.00 | 0.58] | 0.12 | [-0.19 | 0.44] |
| [Education=middle] | -0.03 | [-0.20 | 0.14] | 0.15 | [-0.04 | 0.35] | 0.17 | [-0.07 | 0.40] | 0.09 | [-0.16 | 0.35] |
| 6 |  |  |  |  |  |  |  |  |  |  |  |  |
| [Education=high] |  |  |  |  |  |  |  |  |  |  |  |  |
| Age | -0.00* | [-0.01; | -0.00] | 0.00 | [-0.00 | 0.01] | -0.02*** | [-0.02 | -0.01] | -0.01*** | [-0.02 | -0.01] |
| Z_Att | 0.15*** | [0.09; | 0.22] | -0.11*** | [-0.17 | -0.05] | -0.52*** | [-0.60 | -0.44] | 0.45*** | [0.36 | 0.53] |
| Z_OD | -0.23*** | [-0.29; | -0.16] | -0.04 | [-0.10 | 0.03] | -0.25*** | [-0.34 | -0.17] | -0.12* | [-0.21 | 0.02] |
| Z_SD | -0.25*** | [-0.32; | -0.18] | 0.14*** | [0.08 | 0.21] | 0.21*** | [0.13 | 0.30] | .16*** | [0.07 | 0.26] |
| Z_Att* Z_OD | -0.11*** | [-0.16; | -0.05] | -0.10*** | [-0.15 | -0.06] | -0.16*** | [-0.23 | -0.09] | -0.03 | [-0.10 | 0.05] |
| Z_Att * Z_SD | -0.04 | [-0.10; | 0.02] | .14*** | [0.09 | 0.19] | 0.01 | [-0.06 | 0.08] | 0.10** | [0.02 | 0.17] |
| Z_OD * Z_SD | -0.00 | [-0.05; | 0.06] | -0.05* | [-0.10 | -0.00] | -0.10** | [-0.17 | -0.04] | -0.07† | [-0.14 | 0.00] |

*** *p* < .001, ** *p* < .01, * *p* < .05, † *p* < .10

Table B3.1. Parameter estimates and 95% confidence intervals for the effects of the Polarization index, personal attitude (Att) and their interaction on the DVs in Study 3 (Dutch Sample).

|  | Conversational harmony | | | Avoidance | | | Negative emotions target statement | | | Relationship threat | | | | Incrementality beliefs | | |
| --- | --- | --- | --- | --- | --- | --- | --- | --- | --- | --- | --- | --- | --- | --- | --- | --- |
| Intercept | 3.77*** | [2.44; | 5.10] | 3.96*** | [2.58; | 5.35] | 4.06*** | [2.39; | 5.72] | | 3.94*** | [2.15; | 5.73] | 3.40*** | [1.98; | 4.82] |
| [R = 1] | 0.14* | [0.00; | 0.27] | 0.15* | [0.02; | 0.27] | -0.22** | [-0.37; | -0.07] | | -0.08 | [-0.21; | 0.05] | 0.23*** | [0.10; | 0.36] |
| [R = 2] | -.06 | [-0.20; | 0.07] | 0.04 | [-0.08; | 0.17] | -0.08 | [-0.23; | 0.07] | | -0.01 | [-0.14; | 0.13] | 0.06 | [-0.07; | 0.19] |
| [R = 3] |  |  |  |  |  |  |  |  |  | |  |  |  |  |  |  |
| [C = polarized] | -0.10 | [-0.27; | 0.08] | 0.04 | [-0.12; | 0.20] | -0.10 | [-0.30; | 0.09] | | -0.11 | [-0.28; | 0.07] | -0.07 | [-0.24; | 0.09] |
| [C = dispersed] | -0.09 | [-0.27; | 0.08] | 0.06 | [-0.10; | 0.22] | -0.11 | [-0.31; | 0.09] | | -0.06 | [-0.24; | 0.11] | -0.02 | [-0.19; | 0.14] |
| [C = disagree] | -0.04 | [-0.26; | 0.17] | 0.17 | [-0.03; | 0.37] | -0.10 | [-0.35; | 0.15] | | -0.05 | [-0.27; | 0.17] | 0.01 | [-0.20; | 0.22] |
| [C = agree] |  |  |  |  |  |  |  |  |  | |  |  |  |  |  |  |
| [G =male] | 0.41 | [-0.90; | 1.71] | -0.75 | [-2.12; | 0.61] | -0.23 | [-1.85; | 1.40] | | -0.53 | [-2.30; | 1.24] | 0.55 | [-0.84; | 1.95] |
| [G =female] | 0.24 | [-1.06; | 1.55] | -0.80 | [-2.17; | 0.56] | -0.37 | [-2.00; | 1.26] | | -0.65 | [-2.42; | 1.11] | 0.54 | [-0.86; | 1.94] |
| [G = other] |  |  |  |  |  |  |  |  |  | |  |  |  |  |  |  |
| Age | -0.01* | [-0.01; | -0.00] | 0.01** | [0.00; | 0.01] | -0.01* | [-0.01; | -0.00] | | -0.01* | [-0.01; | -0.00] | -0.01* | [-0.01; | -0.00] |
| Polarization Index | -0.11 | [-0.43; | 0.20] | -0.34* | [-0.65; | -0.04] | -0.58** | [-0.95; | -0.20] | | 0.05 | [-0.29; | 0.39] | 0.35* | [0.04; | 0.67] |
| Z_Att | -0.08 | [-0.19; | 0.03] | 0.02 | [-0.08; | 0.12] | -0.38*** | [-0.51; | -0.25] | | -0.02 | [-0.13; | 0.10] | -0.10 | [-0.20; | 0.02] |
| Polarization Index * Z_Att | 0.30* | [0.03; | 0.56] | 0.08 | [-0.17; | 0.33] | -0.16 | [-0.47; | 0.14] | | -0.02 | [-0.29; | 0.25] | 0.12 | [-0.16; | 0.36] |

*** *p* < .001, ** *p* < .01, * *p* < .05, † *p* < .10

Table B3.2 Parameter estimates and 95% confidence intervals for the effects of subscales Opinion differentiation (OD) and Structural differentiation (SD) of the polarization questionnaire, personal attitudes (Att), and their interactions on each DV in Study 3 (Dutch Sample).

*** *p* < .001, ** *p* < .01, * *p* < .05, † *p* < .10

|  | Conversational harmony | | | Avoidance | | | Negative emotions target statement | | | Relationship threat | | | | Incrementality beliefs | | |
| --- | --- | --- | --- | --- | --- | --- | --- | --- | --- | --- | --- | --- | --- | --- | --- | --- |
| Intercept | 3.66*** | [2.35; | 4.97] | 3.95*** | [2.62; | 5.28] | 3.93*** | [2.27; | 5.55] | | 4.11*** | [2.47; | 5.75] | 3.41*** | [2.11; | 4.68] |
| [R = 1] | 0.19** | [0.06; | 0.33] | 0.17** | [0.05; | 0.30] | -0.22** | [-0.37; | -0.06] | | -0.06 | [-0.19; | 0.08] | 0.20** | [0.07; | 0.33] |
| [R = 2] | -0.06 | [-0.19; | 0.08] | 0.04 | [-0.08; | 0.17] | -0.08 | [-0.23; | 0.07] | | -0.00 | [-0.13; | 0.13] | 0.05 | [-0.08; | 0.18] |
| [R = 3] |  |  |  |  |  |  |  |  |  | |  |  |  |  |  |  |
| [C = polarized] | -0.05 | [-0.23; | 0.12] | 0.04 | [-0.12; | 0.20] | -0.12 | [-0.32; | 0.08] | | -0.10 | [-0.27; | 0.07] | -0.08 | [-0.24; | 0.09] |
| [C = dispersed] | -0.04 | [-0.21; | 0.13] | 0.07 | [-0.09; | 0.23] | -0.10 | [-0.30; | 0.10] | | -0.06 | [-0.23; | 0.11] | -0.04 | [-0.20; | 0.13] |
| [C = disagree] | -0.02 | [-0.24; | 0.19] | 0.19† | [-0.01; | 0.38] | -009 | [-0.34; | 0.16] | | -0.07 | [-0.28; | 0.15] | 0.01 | [-0.20; | 0.21] |
| [C = agree] |  |  |  |  |  |  |  |  |  | |  |  |  |  |  |  |
| [G =male] | 0.39 | [-0.90; | 1.67] | -0.88 | [-2.19; | 0.44] | -0.26 | [-1.85; | 1.34] | | -0.67 | [-2.30; | 0.95] | 0.66 | [-0.62; | 1.95] |
| [G =female] | 0.25 | [-1.04; | 1.53] | -0.88 | [-2.19; | 0.43] | -0.36 | [-1.95; | 1.23] | | -0.73 | [-2.36; | 0.89] | 0.59 | [-0.70; | 1.88] |
| [G = other] |  |  |  |  |  |  |  |  |  | |  |  |  |  |  |  |
| Age | -0.01* | [-0.01; | -0.00] | 0.01*** | [0.00; | 0.01] | -0.01* | [-0.01; | 0.00] | | -0.01** | [-0.01; | -0.00] | -0.00† | [-0.01; | 0.00] |
| Z_Att | 0.02 | [-0.05; | 0.08] | 0.02 | [-0.04; | 0.08] | -0.46*** | [-0.53; | -0.38] | | -0.05 | [-0.12; | 0.01] | -0.02 | [-0.08; | 0.04] |
| Z_OD | -0.14*** | [-0.21; | -0.07] | -0.15*** | [-0.22; | -0.09] | -0.14** | [-0.22; | -0.05] | | -0.23*** | [-0.30; | -0.16] | 0.28*** | [0.21; | 0.34] |
| Z_SD | -0.15*** | [-0.22; | -0.08] | 0.14*** | [0.08; | 0.20] | 0.16*** | [0.08; | 0.24] | | 0.30*** | [0.23; | 0.38] | -0.24*** | [-0.31; | -0.18] |
| Z_Att * Z_OD | -0.01 | [-0.06; | 0.05] | -0.08** | [-0.14; | -0.03] | -0.06† | [-0.13; | 0.01] | | -0.04 | [-0.10; | 0.01] | -0.03 | [-0.09; | 0.06] |
| Z_Att * Z_SD | -0.00 | [-0.06; | 0.06] | -0.01 | [-0.06; | 0.05] | -0.02 | [-0.05; | 0.08] | | -0.07* | [-0.01; | 0.13] | -0.00 | [-0.06; | 0.05] |
| Z_OD * Z_SD | -0.01 | [-0.06; | 0.05] | -0.04 | [-0.09; | 0.01] | -0.01 | [-0.05; | 0.07] | | -0.00 | [-0.05; | 0.06] | -0.01 | [-0.06; | 0.05] |

*** *p* < .001, ** *p* < .01, * *p* < .05, † *p* < .10

**Appendix C. Analyses including interaction effects for all three studies.**

In Study 3, four serial indirect effect models served to examine whether our manipulation would increase perceived opinion differentiation, which would in turn decrease relational threat (1) while increasing incrementality beliefs (2), and through that, would decrease avoidance and decrease negative emotions. We also teste whether our manipulation would increase structural differentiation, which in turn would increase relational threat (3) while decreasing incrementality beliefs (4), and through that, would increase avoidance and increase negative emotions. Model 1 and 2 included structural differentiation as a covariate, and Model 3 and 4 included opinion differentiation as a covariate. The models are displayed in Figure C1-2. All four serial mediations were tested with PROCESS Macro model 6 (Hayes, 2004), and did not correct for the interdependence of the data within participants. We therefore advice that conclusions from these analyses are drawn with caution.

Figure C1. Indirect effects of manipulation via Opinion Differentiation and Structural differentiation, and through Incrementality of beliefs on the conversation consequences.


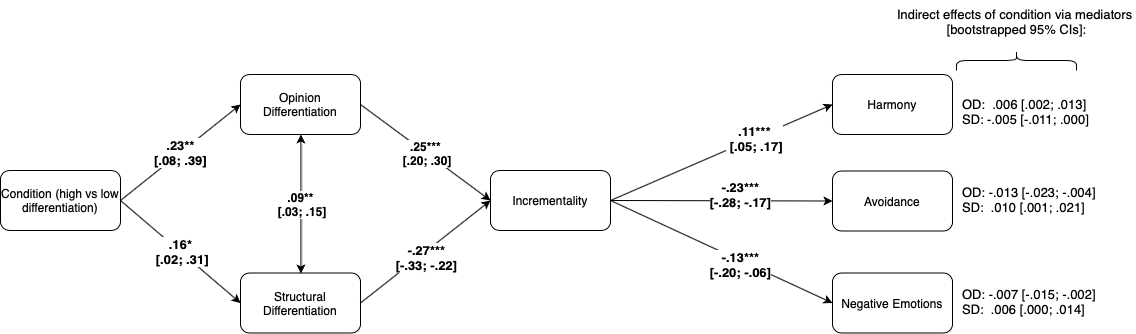


Figure C2. Indirect effects of manipulation via Opinion Differentiation and Structural differentiation, and through Relational Threat on the conversation consequences.


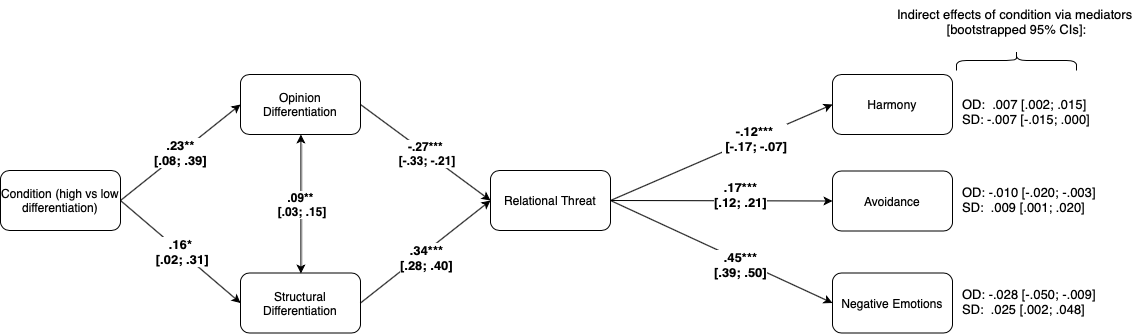

Supplement: sj-docx-2-psp-10.1177_01461672211030816 – Supplemental material for A Polarized Discourse: Effects of Opinion Differentiation and Structural Differentiation on Communication [file sj-docx-2-psp-10.1177_01461672211030816.docx]
